# An Evolutionary Analysis of Antigen Processing and Presentation across Different Timescales Reveals Pervasive Selection

**DOI:** 10.1371/journal.pgen.1004189

**Published:** 2014-03-27

**Authors:** Diego Forni, Rachele Cagliani, Claudia Tresoldi, Uberto Pozzoli, Luca De Gioia, Giulia Filippi, Stefania Riva, Giorgia Menozzi, Marta Colleoni, Mara Biasin, Sergio Lo Caputo, Francesco Mazzotta, Giacomo P. Comi, Nereo Bresolin, Mario Clerici, Manuela Sironi

**Affiliations:** 1 Scientific Institute IRCCS E. MEDEA, Bioinformatics, Bosisio Parini, Italy; 2 Department of Biotechnology and Biosciences, University of Milan-Bicocca, Milan, Italy; 3 Department of Biomedical and Clinical Sciences, University of Milan, Milan, Italy; 4 Infectious Disease Unit, S. Maria Annunziata Hospital, Florence, Italy; 5 Dino Ferrari Centre, Department of Physiopathology and Transplantation, University of Milan, Fondazione Ca' Granda IRCCS Ospedale Maggiore Policlinico, Milan, Italy; 6 Chair of Immunology, Department of Physiopathology and Transplantation, University of Milan, Milan, Italy; 7 Don C. Gnocchi Foundation ONLUS, IRCCS, Milan, Italy; University of Colorado School of Medicine, United States of America

## Abstract

The antigenic repertoire presented by MHC molecules is generated by the antigen processing and presentation (APP) pathway. We analyzed the evolutionary history of 45 genes involved in APP at the inter- and intra-species level. Results showed that 11 genes evolved adaptively in mammals. Several positively selected sites involve positions of fundamental importance to the protein function (e.g. the TAP1 peptide-binding domains, the sugar binding interface of langerin, and the CD1D trafficking signal region). In CYBB, all selected sites cluster in two loops protruding into the endosomal lumen; analysis of missense mutations responsible for chronic granulomatous disease (CGD) showed the action of different selective forces on the very same gene region, as most CGD substitutions involve aminoacid positions that are conserved in all mammals. As for ERAP2, different computational methods indicated that positive selection has driven the recurrent appearance of protein-destabilizing variants during mammalian evolution. Application of a population-genetics phylogenetics approach showed that purifying selection represented a major force acting on some APP components (e.g. immunoproteasome subunits and chaperones) and allowed identification of positive selection events in the human lineage.

We also investigated the evolutionary history of APP genes in human populations by developing a new approach that uses several different tests to identify the selection target, and that integrates low-coverage whole-genome sequencing data with Sanger sequencing. This analysis revealed that 9 APP genes underwent local adaptation in human populations. Most positive selection targets are located within noncoding regions with regulatory function in myeloid cells or act as expression quantitative trait loci. Conversely, balancing selection targeted nonsynonymous variants in *TAP1* and *CD207* (langerin). Finally, we suggest that selected variants in *PSMB10* and *CD207* contribute to human phenotypes. Thus, we used evolutionary information to generate experimentally-testable hypotheses and to provide a list of sites to prioritize in follow-up analyses.

## Author Summary

Antigen-presenting cells digest intracellular and extracellular proteins and display the resulting antigenic repertoire on cell surface molecules for recognition by T cells. This process initiates cell-mediated immune responses and is essential to detect infections. The antigenic repertoire is generated by the antigen processing and presentation pathway. Because several pathogens evade immune recognition by hampering this process, genes involved in antigen processing and presentation may represent common natural selection targets. Thus, we analyzed the evolutionary history of these genes during mammalian evolution and in the more recent history of human populations. Evolutionary analyses in mammals indicated that positive selection targeted a very high proportion of genes (24%), and revealed that many selected sites affect positions of fundamental importance to the protein function. In humans, we found different signatures of natural selection acting both on regions that are expected to regulate gene expression levels or timing and on coding variants; two human selected polymorphisms may modulate the susceptibility to Crohn's disease and to HIV-1 infection. Therefore, we provide a comprehensive evolutionary analysis of antigen processing and we show that evolutionary studies can provide useful information concerning the location and nature of functional variants, ultimately helping to clarify phenotypic differences between and within species.

## Introduction

Cell mediated immune responses are initiated by the recognition of an MHC/antigen complex on the surface of an APC (antigen presenting cell) by a T cell receptor (TcR). MHC class I and II molecules present peptides to T cells that express the CD8 or CD4 molecules, respectively.

Non-conventional T cell populations also exist that express TcRs with semi-invariant α-chains: MAIT (mucosal-associated invariant T) cells recognize antigens bound to the class Ib MHC molecule MR1, and iNKT (invariant natural killer T) cells respond to lipids and glycolipid antigens bound to CD1D.

**Figure 1:**
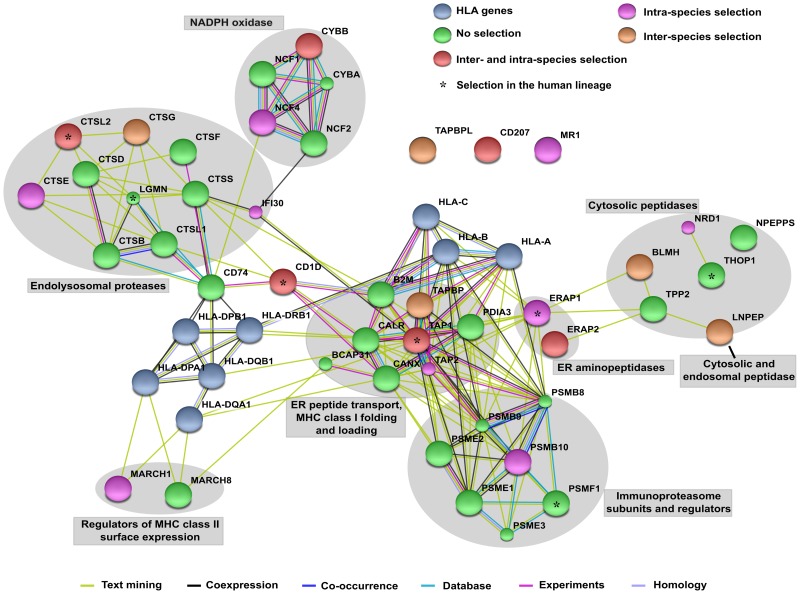
STRING interaction diagram of the analyzed genes. Classic *HLA* class I and class II genes are also shown (although not analyzed). Each filled node denotes a gene; edges between nodes indicate interactions between protein products of the corresponding genes. Different edge colors represent the types of evidence for the association. Annotation of genes and gene clusters refers to their major activity/location in APP. Genes are colored according to the observed selection signatures either described herein or in previous works [33], [43], [44].

Whatever the nature of the presenting molecule, the limited dimension of its cleft makes it impossible for macromolecules to be presented: only fragments deriving from the lysis of such molecules will be nested in the cleft. Most steps leading to the formation of MHC class I- and II-peptide complexes have been defined [1]. Peptides that will be embedded into the cleft of class I molecules are initially processed by the proteasome, a complex structure located in the cytoplasm. Immune cells and other cell types exposed to interferon gamma express a variant of the proteasome referred to as the immunoproteasome and differing in a few subunit components (Figure 1) [1]. The proteasome activity can be complemented in the cytosol by endopeptidases (Figure 1) [1], [2]. Channels formed by TAP molecules (TAP1 and TAP2) allow peptides generated in the cytoplasm to be transported into the endoplasmic reticulum (ER), where they may be trimmed at their N-terminal end by ERAP proteins. In the ER, MHC class I are bound to the TAP complex through tapasin (TAPBP), and they are further stabilized by two chaperones, calreticulin (CALR) and ERp57 (PDIA3) [1] (Figure 1). The whole complex is referred to as the peptide-loading complex (PLC). The peptide/MHC class I dimer will then bind a molecule of β2 microglobulin; this results in the stabilization of the complex that will be exported to the cell surface by an exocytic vescicle [1].

MHC class II molecules wait for the proper peptide in endosomes; these will fuse with lysosomes where the exogenous proteins have been processed by resident proteases (Figure 1). The removal of the CD74-derived invariant DM peptide by cathepsin S or L (CTSS, CTSL1) from the cleft of the MHC molecule will render it available to the incoming peptides. The resulting MHC/peptide complexes will then be exported to the cell surface by endosomes [1].

Finally, in cross-presentation phagocytosed antigens are partially degraded, exported to the cytoplasm for further processing, and then loaded onto MHC class I molecules. A central role in this process is played by the superoxide-producing phagocyte NADPH-oxidase, a multiprotein complex (Figure 1) which regulates alkalinization of the phagosomal lumen [3].

Classic MHC molecules are encoded by genes that show extreme levels of polymorphism in most vertebrates and several studies have demonstrated that diversity at the peptide binding region is maintained by natural selection [4]. Thus, their role in adaptive immunity and their pattern of diversity indicate adaptation to a wide range of pathogen species leading to aminoacid diversification of the antigen binding cleft. Nonetheless, the generation and loading of the antigenic repertoire presented by MHC molecules also depend on the action of a number of molecules, as detailed above. Therefore, it is straightforward to imagine that a proportion of these should be targeted by natural selection, as well. The observation whereby several pathogens encode molecules that hijack specific components of the antigen processing and presentation (APP) pathway further supports this possibility [5]. Herein, we investigated the evolutionary history of 45 genes with a central role in APP by analyzing inter-specific divergence in mammals and intra-specific diversity in human populations.

## Results

### Several APP genes evolved adaptively in mammals

To analyze the evolutionary history of the APP pathway, we compiled a list of 45 genes that play roles of central importance in this process. Specifically, based on Gene Ontology classification, we included genes involved in the processing of both endogenous and exogenous antigens and in the presentation via class I, class II or class Ib MHC molecules (see methods for details of gene selection criteria) (Figure 1, Supplementary Table S1). Because they have already been the topic of extensive investigation, *HLA* genes were not included. Moreover, genes involved in APP, but also in general cellular processes (e.g. components of the constitutive proteasome, genes involved in vesicle trafficking) were not analyzed.

First we analyzed the evolutionary history of these genes in mammals by retrieving coding sequence information for all available species. For *CTSL1* and *CTSL2* only primate sequences were included because the two genes originated from a relatively recent duplication event (which occurred before the split of modern primates) and, due to their high similarity, it is very difficult to establish one-to-one orthology with more distantly related mammals.

Analysis of sequence alignments revealed that all genes evolved under purifying selection, as the average non-synonymous substitution rate (dN) was generally lower than the rate for synonymous substitutions (dS) (Supplementary Table S2). Yet, positive selection can operate on specific residues or domains within coding regions that are otherwise selectively constrained. To test this possibility we applied maximum-likelihood analyses by comparing models of gene evolution that allow (NSsite models M2a and M8) or disallow (NSsite models M1a, and M7) a class of codons to evolve with dN/dS>1 [6]. After accounting for the presence of recombination (that might yield false positive results [7]) and using different models of codon frequency (see Materials and Methods and Supplementary Figure S1), eleven APP genes (*BLMH*, *CD1D*, *CD207*, *CTSL2*, *CTSG*, *CYBB*, *ERAP2*, *LNPEPS*, *TAPBP*, *TAPBPL*, and *TAP1*) were found to evolve adaptively in mammals (Table 1, Figure 1, Supplementary Table S3 and S4). To identify specific sites subject to positive selection, we applied two methods: the Bayes Empirical Bayes (BEB) analysis (with a cut-off of 0.90) from M8 [8], and the Mixed Effects Model of Evolution (MEME) (with the default cutoff of 0.1) [9]. Only sites detected using both methods were considered and these are listed in Table 1.

**Table 1:** Evolutionary analysis of mammalian/primate APP genes.

Gene (length in codons)^a^	N species^b^	N recombination breakpoints^c^	N significant regions^d^	Positively selected sites (human codons)^e^
***BLMH*** (455)	39	2	1	211V, 388A, 390T
***CD207*** (329)	32	0	1	213P, 289A
***CD1D*** (353)	28	1	2	25L, 108L, 136F, 139K, 157L, 161L, 302M, 322T
***CTSG*** (255)	28	0	1	66W, 69N, 106Q, 122R, 177G, 221S
***CTSL2*** (334)	11	0	1	262S
***CYBB*** (570)	38	2	1	136P, 148Q, 149N, 233A, 234E, 237A, 240N, 241I, 242T, 243V, 245E, 249S, 250E, 255K
***ERAP2*** (970)	26	2	1	416Y, 420V, 857A
***LNPEP*** (1025)	38	1	1	872K, 884I, 918N, 1023W
***TAP1*** (777)	35	1	2	R137, E145, G225, Q516, L557, L562
***TAPBP*** (468)	33	0	1	67S, 225N
***TAPBPL*** (438)	32	0	1	394G, 433T

a Only genes subject to positive selection (see text) are shown.

b Number of species in the alignment.

c Number of recombination breakpoints from GARD.

d Number of gene regions showing evidences of positive selection (see text).

e Positively selected sites identified by both BEB and MEME.

**Figure 2:**
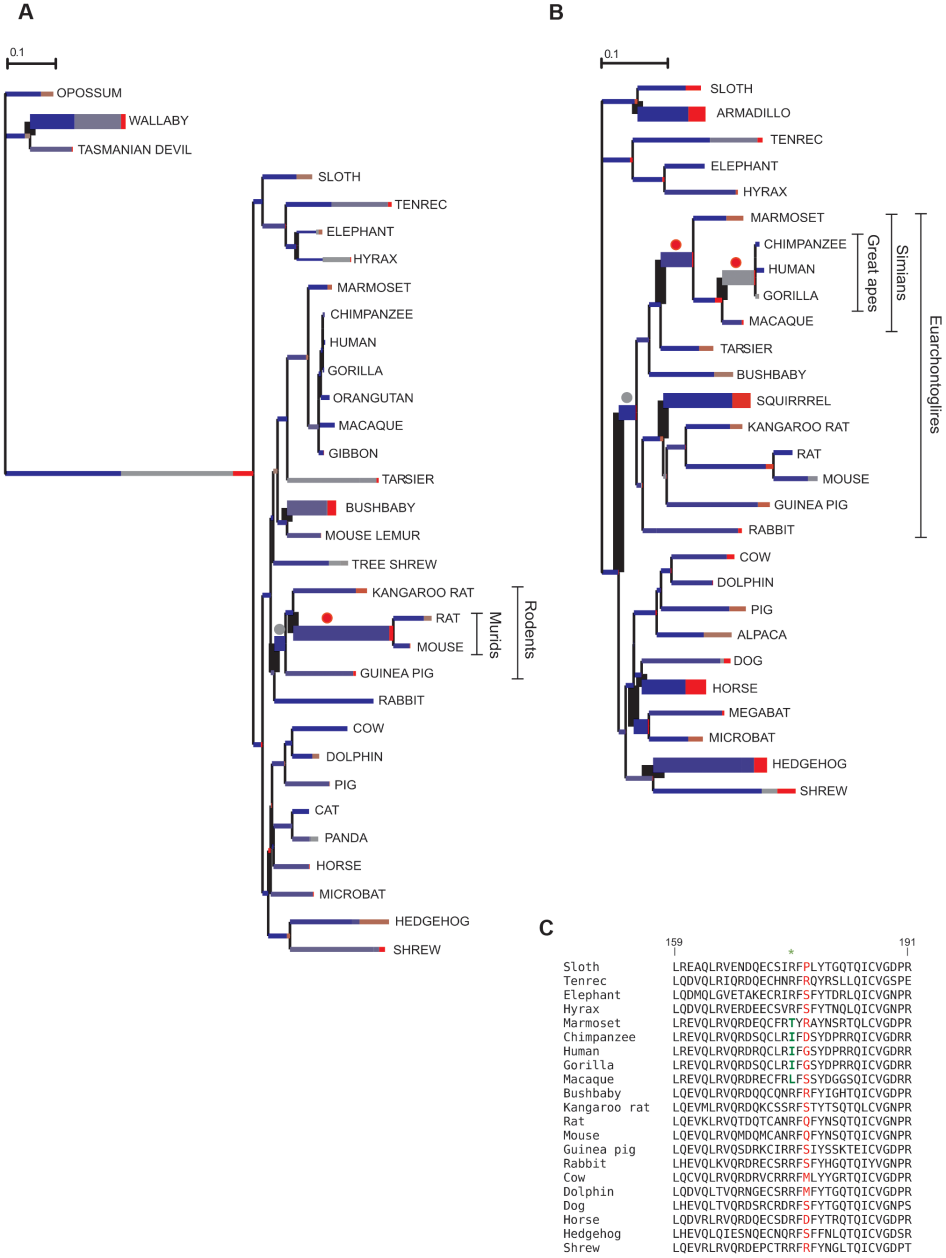
Branch-site analysis of positive selection for *CD207* and *CTSG*. BS-REL analysis for *CD207* (A) and *CTSG* (B). Branch lengths are scaled to the expected number of substitutions per nucleotide, and branch colors indicate the strength of selection (dN/dS or ω). Red, positive selection (ω>5); blue, purifying selection (ω = 0); grey, neutral evolution (ω = 1). The proportion of each color represents the fraction of the sequence undergoing the corresponding class of selection. Thick branches indicate statistical support for evolution under episodic diversifying selection as determined by BS-REL. Dots denote branches that were confirmed (red) or not (gray) to be under positive selection using the PAML branch-site models (after FDR correction for multiple tests). (C) Alignment of a portion of the CTSG peptidase domain for a few representative mammals showing positively selected residues in simians (green) and in the whole phylogeny (red).

In order to explore possible variations in selective pressure among different lineages, we used the branch site-random effects likelihood (BS-REL) method [10], which was applied to the 45 APP gene alignments or to sub-regions (alignments were split on the basis of recombination breakpoint location). BS-REL makes no a priori assumption about which lineages are more likely to represent selection targets. We focused our attention on genes showing evidences of episodic positive selection in lineages that include the human species (i.e. the human lineage or branches leading to great apes) or in branches leading to murids (due to the relevance these species have as model organisms). Thus, three genes were selected for further analysis: *CD207*, *CTSG*, and *CYBB* (Figure 2 and Supplementary Figure S2). For these alignments, the primate/murid branches detected by BS-REL were cross-validated using the branch-site models implemented in PAML [11], which apply a likelihood ratio test to compare a model (MA) that allows positive selection on one or more lineages (foreground lineages) with a model (MA1) that does not allow such positive selection. As suggested [12], a false discovery rate (FDR) correction was applied to these p values, as multiple hypotheses are being tested on the same phylogeny. As shown in figure 2, PAML confirmed episodic positive selection at 1 and 2 branches in *CD207* and *CTSG*, respectively; no *CYBB* branch was validated by PAML (Supplementary Table S5, Supplementary Figure S2). The PAML branch-site models can identify specific sites that evolved under positive selection in the foreground branches; this is achieved through implementation of a BEB analysis, which is accurate but has low statistical power [11]. BEB analysis identified one positively selected site in CTSG (175I) on the lineage leading to simians.

In line with its ability to detect episodic positive selection, the MEME analysis performed on the whole phylogeny also detected the 175I residue in CTSG. Thus, episodic positive selection acted on *CTSG* and *CD207* in simians and murids, respectively.

### Positively selected sites involve functional residues

We next analyzed the location of positively selected sites relative to known protein domains or crystal structures.

**Figure 3:**
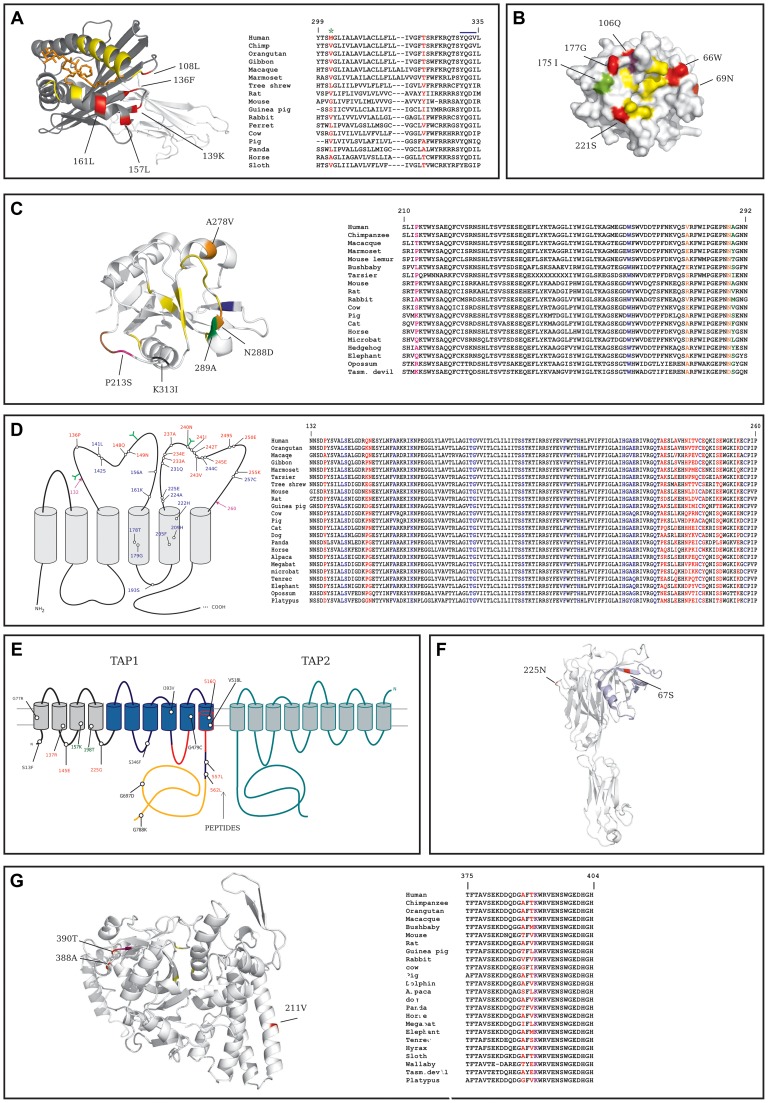
Analysis of positively selected sites. In all panels aminoacid numbering refers to the human protein. (A) Left: ribbon diagram of the extracellular domain of human CD1D bound to α-galactosylceramide (orange). Positively selected sites are shown in red, the α1/α2 and α3 domains are depicted in dark and light grey, respectively. Yellow residues form the contact interface with the TcR. Right: alignment of the transmembrane and cytoplasmic domains of CD1D for a few representative mammals; positively selected sites are in red and the YxxZ sequence is marked (blue line); the green asterisk denotes a site positively selected in the human lineage. (B) Surface structure of the protease domain of human CTSG; sites that define substrate binding pockets or form the catalytic triad are shown in yellow; positively selected sites are in red (whole phylogeny) and green (simians). The violet residue confers to CTSG the ability to cleave Shigella virulence factors if mutated. 126R is not visible as it is located on the back surface. (C) Left: ribbon diagram of the human CD207 CRD. Color codes are as follows: yellow, sites directly involved in sugar binding; green, positively selected site at the sugar binding interface; brown, sites involved in trimer formation; orange, nonsynonymous SNPs; magenta, positively selected site that is polymorphic in humans; black, missense SNP at the sugar binding interface; blue, a human mutation responsible for Birbeck granule deficiency. Right: alignment of a portion of the CRD for a few representative mammals; color codes are as in the left panel. (D) Positively selected sites for CYBB are shown relative to the membrane topology (left); sites subject to diversifying selection are in red, mutations responsible for CGD or MSMD are in blue (note that mutations are shown only if falling in the region where positively selected sites are located); glycosylation sites are represented in green; the magenta arrows denote the region which is represented in the multiple species alignment (right, color codes as in the membrane topology diagram). (E) Membrane topology arrangement and positively selected sites for TAP1; TAP2 (green profiled) is shown although no positively selected sites were identified. The TAP1 unique N-terminal domain is shown as grey cylinders, the ABC transporter domain is in blue; the nucleotide binding domain is in orange and the protein portions that bind peptides are profiled in red. Sites subject to diversifying selection are in red, human missense polymorphisms in black, positively selected sites in the human lineage are in green. (F) Ribbon diagram of human tapasin; positively selected sites are shown in red; the 87 N-terminal aminoacids that facilitate the folding of MHC I-peptide complexes are in light blue. (G) Left: ribbon diagram of human BLMH (one subunit of the hexameric complex is shown); positively selected sites are in red, the acetylated/ubiquitinated lysine (391K) is in violet, the catalytic triad in yellow. Right: alignment of the region surrounding 391K and two positively selected sites for a few representative mammals; color codes as in the left panel.

The extracellular portion of CD1D comprises two domains (α1 and α2) that form the antigen-binding groove and interact with the TcR, plus an α3 domain that interacts with B2M. All positively selected sites we identified in the extracellular portion of the protein are in the α1/α2 domains, and four of them cluster in a spatially defined region in the C' pocket; these positions are not directly involved in the binding of known antigens, and one of them flanks the TcR interaction surface (Figure 3A). One additional positively selected site was located in the short CD1D cytoplasmic tail, which carries signals essential for CD1D cellular trafficking. Specifically, the human 322T residue is essential for transportation to the plasma membrane [13].

*CD207* encodes langerin, a C-type lectin that binds glycoconjugates and functions as a trimer [14], [15]. The extracellular portion of the protein contains a carbohydrate-recognition domain (CRD) and a neck region that participates in trimer formation. The two positively selected sites are located in the CRD domain; one of them (289A) is directly involved in Ca^+^ mediated carbohydrate binding [14] (Figure 3C); the other site (213P) immediately flanks residues that contribute to the interaction among langerin subunits forming the trimer. The W264R mutation in *CD207* has been associated with Birbeck granule deficiency [16] and 264W is conserved in all mammals (Figure 3C).

The *CYBB* gene encodes an integral membrane protein that functions as the catalytic subunit of the phagocyte NADPH oxidase. Because the crystallographic structure of CYBB has not been solved, we mapped selected sites onto the membrane topology arrangement [17]: results indicated that all sites are located in extracellular/phagosome lumenal loops; specifically several sites cluster in the third loop and one of these (240N) affects a glycosylation site [18]. *CYBB* mutations are responsible for X-linked chronic granulomatous disease (CGD) [19] and for mendelian inheritance to mycobacterial diseases (MSMD) [20]; analysis of MSMD and CGD missense mutations located in the region where the positively selected sites were detected indicated that they all affect extremely conserved positions (Figure 3D).

TAP1 and TAPBP (tapasin) are part of the PLC. TAP1 belongs to the family of ATP binding cassette (ABC) transporters and its membrane topology has been determined [21]. Three of the positively selected sites we identified are located in the transmembrane region or cytoplasmic loops of the TAP1 unique N-terminal domain that is involved in the binding of tapasin (TAPBP) [22]. Interestingly, three additional sites subject to diversifying selection are located within or very close to the pore-forming region of TAP1 - i.e. the region responsible for peptide binding and transportation (Figure 3E) [23]. As for tapasin, one of the two positively selected sites is directly involved in ERp57 binding (225N) [24] and the second one is located at the N-terminus (67S) (Figure 3F). The cystein residue involved in disulfide-bonding with PDIA3 is conserved in all eutheria but not in metatheria (Supplementary Figure S3).

Positively selected sites were also identified in two cathepsin family members whose crystal structure has been solved. In CTSG the six sites subject to pervasive positive selection are located within the serine protease domain and three of them immediately flank (66W and 221S) or overlap (177G) residues that define the substrate binding pockets [25] (Figure 3B). This also applies to the 175I residue, targeted by positive selection in the simian lineage (Figure 3B).

As for CTSL2, one positively selected site was found in the protease domain, outside the substrate binding pockets (Supplementary Figure S3).

*LNPEP* encodes leucyl/cystinyl aminopeptidase; the four positively selected sites were found to be located in the C-terminal domain 4, which has been shown to possess regulatory activity [26] (Supplementary Figure S3).

Three sites subject to diversifying selection were also detected in *BLMH*, which encodes a cytoplasmic cysteine protease highly conserved from yeast to mammals [27]. One of them (211V) is located on an exposed α-helix (Figure 3G); the other two sites are on an unstructured loop and immediately flank a lysine residue (391K) which undergoes acetylation and ubiquitination [28], [29]. The modified lysine and most aminoacids in the region are highly conserved, including a phenylalanine at position −2 relative to 391K that is present in all eutheria (Figure 3G) and represents a highly preferred residue in cytosolic acetylation sites [29].

**Figure 4:**
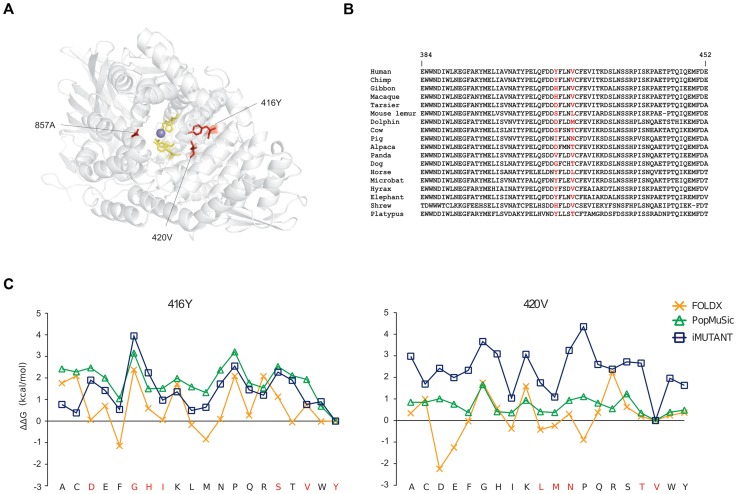
Analysis of positively selected sites in *ERAP2*. (A) Ribbon diagram of ERAP2: positively selected sites are shown in red and those that coordinate the Zn ion (violet) in yellow. (B) Alignment of the region surrounding 416Y and 420V (in red) for a few representative mammals. (C) ΔΔG in kcal/mol for 416Y (left), 420V (right) mutations to all other 19 residues of the ERAP2 structure or sequence; results are shown for FoldX, PopMuSiC, and I-Mutant.

Finally, in ERAP2 we identified three positively selected sites, which seem not be involved in proteolytic activity. 3D-structure protein analysis indicated that the three residues are located on α helices shaping the internal cavity of the protein where the catalytic Zn ion is coordinated (Figure 4A). Two of these residues are involved in several short-range interactions: 416Y can interact hydrofobically with 362L, 413F, 746W, and 420V (and *vice-versa*); the same kind of interactions can be made by 420V with 417F (not shown); a side-chain side-chain H-bond can be formed by the OH group of 416Y and the NH_2_ group of 366R (not shown). Thus, we performed a stability analysis: 416Y and 420V were mutated to all other residues through the use of three different methods. The tyrosine and valine at positions 416 and 420 are the most common aminoacids among the species we analyzed (Figure 4B) and represent the ancestral state residues (see Materials and methods). As shown in Figure 4C, the replacement of the two aminoacids led to changes of different magnitude in ΔG. Although the three programs yielded different ΔΔG values for every mutated residues, the trend was maintained (in particular between I-Mutant and PoPMuSiC) and indicated that replacement of 416Y and 420V with any other aminoacid likely results in protein destabilization (i.e. positive ΔΔG values) (Figure 4C). These observations suggest that positive selection might have driven the recurrent appearance of destabilizing variants in ERAP2.

### Different evolutionary scenarios for APP genes in the human lineage

We next applied a recently developed population genetics-phylogenetics approach to study the evolution of APP genes in the human species. Specifically, we used the gammaMap program [30], that jointly uses intra-specific variation and inter-specific diversity to estimate the distribution of fitness effects (DFE) (i.e. selection coefficients, γ) along coding regions. To this aim, we exploited data from the 1000 Genomes Pilot project deriving from the low-coverage whole genome sequencing of 179 individuals with different ancestry: Europeans (CEU), Yoruba from Nigeria (YRI), and East Asians (AS; Japanese plus Chinese) [31]. Ancestral sequences were reconstructed by parsimony from the human, chimpanzee, orangutan and macaque sequences. We noted that no human variant mapped to *NCF1* in CEU and AS. Inspection of accessibility by pair-end next generation sequencing approaches (see Materials and Methods) indicated that *NCF1* is poorly covered in the 1000 Genomes Project data, possibly because of the presence of segmental duplications. We thus discarded genes with less than 80% of accessible sequence; this resulted in the removal of *NCF1* and *NPEPPS*, which were excluded from further analyses.

**Figure 5:**
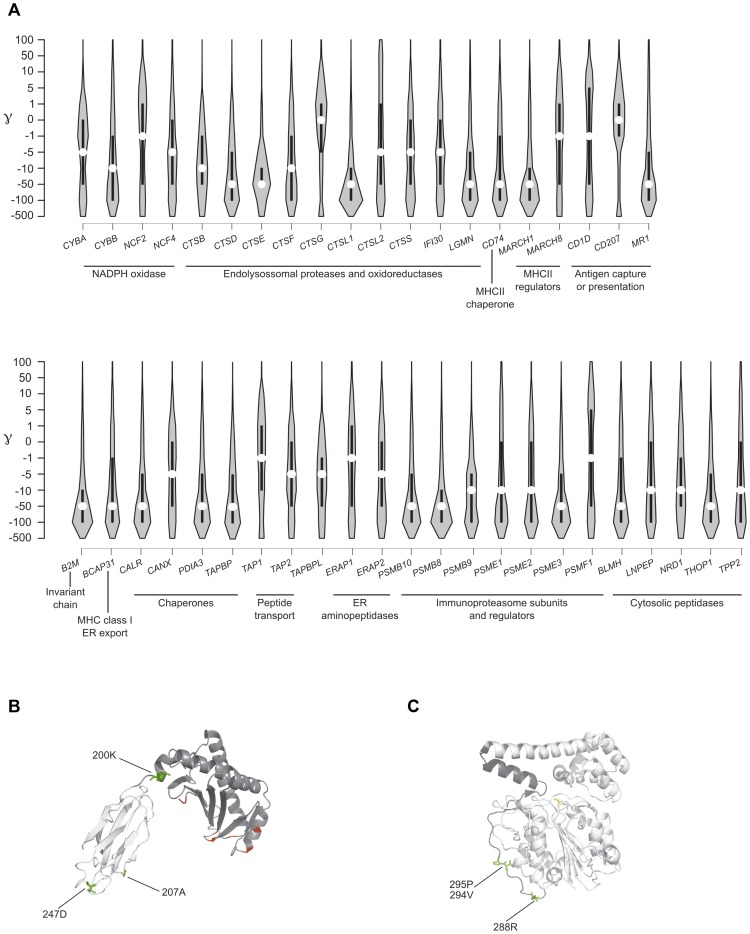
Analysis of selective pressure in the human lineage for APP genes. (A) Violin plot of selection coefficients for APP genes (median, white dot; interquartile range, black bar). Selection coefficients (γ) are classified as strongly beneficial (100, 50), moderately beneficial (10, 5), weakly beneficial (1), neutral (0), weakly deleterious (−1), moderately deleterious (−5, −10), strongly deleterious (−50, −100), and inviable (−500). (B) Ribbon diagram of CD1D; the α1/α2 and α3 domains are depicted in dark and light grey, respectively. Positively selected sites in humans are in green; in red sites selected in the whole phylogeny. (C) Ribbon diagram of LGMN with the activation peptide in dark grey. Human positively selected sites are in green.

We first analyzed the overall distribution of selection coefficients along the 43 APP genes. We observed a general preponderance of codons evolving under negative selection (γ<0) in all APP genes, with few exception including *CD1D*, *CD207*, *CTSG*, and *PSMF1* (Figure 5). The strongest level of negative selection was evident for genes encoding chaperones or proteins involved in MHC class I binding and transport, as well as for loci encoding immunoproteasome subunits. Likewise several endolysosomal proteases and peptidases located in the cytosol showed considerable levels of negative selection (Figure 5).

GammaMap also allows to identify specific codons evolving under positive selection. Herein we defined positively selected codons as those having a cumulative probability >0.80 of γ≥1. Some of these residues had previously been identified in the positive selection analysis we conducted on the whole mammalian phylogeny (Table 2). For example, the 302M residue in CD1D had been detected by both MEME and BEB. Additional selected sites were identified in human CD1D. Among these, residue 200 is at the end of an α-helix that connects domains α1/α2 with α3; this position is occupied by a negatively charged aminoacid in all analyzed primates and mammals (not shown), but the human protein carries a lysine (Figure 5). Likewise, two of the positively selected sites in LGMN were also detected by MEME (Table 2): they are located in the activation peptide (which needs to be removed to generate catalytically active LGMN); in particular, 288R involves the alpha-cleavage site (^287^KRK^289^) [32] (Figure 5). In ERAP1 one of the positively selected sites (R528K, rs30187) has previously been described as a target of balancing selection in human populations [33] (Supplementary Figure S3). Analysis of TAP1 selected sites indicated that they are located in the tapasin binding region, where three sites positively selected in mammals are also observed (Figure 3). As for PSMF1, two positively selected sites map to the N-terminal PI31 proteasome regulator and flank a highly conserved motif important for protein structure [34] (Figure 5). Finally, in THOP1, one of the identified residues is an exposed cystein, which might be involved in multimerization [35] (Supplementary Figure S3).

**Table 2:** Positively selected sites in the human lineage.

Gene	Codon	Ancestral AA	Human AA	dbSNP	Frequency^a^ (YRI; CEU; AS)	Pr^b^	Other methods	Domain/region
*CD1D*	270	Ala	Val	-		0.909	-	Alpha3
	247	Asp	Gly	-		0.893	-	Alpha3
	302	Val	Met	-		0.859	MEME e BEB	Cytoplasmic tail
	200	Glu	Lys	-		0.816	-	Alpha 2
*CTSL2*	207	Met	Val	-		0.942	-	peptidase
*ERAP1*	515	Leu	Val	-		0.912	MEME	Domain II
	528	Lys	Arg	rs30187	0.585; 0.688; 0.514	0.858	-	Domain III
*LGMN*	294	Ile	Val	-		0.981	MEME	activation peptide
	295	Ser	Pro	-		0.977	-	activation peptide
	288	His	Arg	-		0.955	MEME	activation peptide
*PSMF1*	36	Tyr	Cys	rs1803415	0.79; 0.818; 0.448	0.949	-	PI31 proteasome regulator
	18	Thr	Arg	-		0.938	-	PI31 proteasome regulator
	203	His	Pro	-		0.916	MEME	Proline-rich
	192	Ala	Val	rs79465651	0.943; 1; 1	0.914		Proline-rich
*TAP1*	198	Ser	Thr	-		0.867	-	Transmembrane IV
	157	Glu	Lys	-		0.822	-	Transmembrane III
*THOP1*	350	Arg	Cys	rs148139735	0.994; 1^c^	0.971	-	Peptidase
	333	His	Arg	-		0.964	-	Peptidase

a Frequencies derive fron the 1000 Genomes Phase I data.

b Posterior probability of γ>0 as detected by gammaMap.

c These frequencies derive from the NHLBI Exome Sequencing Project (ESP) in African American and European Americans, respectively.

### Natural selection at APP genes is widespread in human populations

To investigate the evolutionary pattern of APP genes during the more recent history of human populations, we again exploited data from the 1000 Genomes Pilot project. A work-flow of the methods we applied is available as Supplementary Figure S4. Briefly, we integrated different neutrality tests that rely on distinct signatures left by natural selection. Thus, over whole gene regions we calculated: 1) θ_W_ [36] and π [37], which describe genetic diversity; 2) Tajima's D [38], normalized Fay and Wu's H [39], as well as Fu and Li's F* and D* [40], which represent site frequency spectrum (SFS)-based statistics. Also, for all SNPs located within APP genes we calculated F_ST_ [41], a measure of population genetic differentiation in pairwise comparisons (CEU/YRI, YRI/AS, and AS/CEU), and we performed the DIND (Derived Intra-allelic Nucleotide Diversity) test [42], which is based on haplotype homozygosity.

Because the low-coverage 1000 Genomes data suffer from a bias in the SFS [31], and in order to account for the influence of human demographic history, we applied an outlier approach by deriving empirical distributions of the same parameters calculated for a randomly selected set of human genes (see Materials and methods).

Analysis of θ_W_ and π for APP genes indicated that 8 of them had values higher than the 95^th^ percentiles in at least one population (Supplementary Figure S5); after excluding *ERAP1*, *ERAP2*, and *TAP2*, which have previously been described as selection targets [33], [43], [44], these genes were considered as balancing selection candidates and were Sanger-resequenced, as detailed below.

For the remaining genes, we investigated whether they have been targets of selective sweeps. To minimize the identification of false positive signals, APP genes were considered targets of directional selection if they represented outliers (in the 5% tails of empirical distributions) in the same population for at least three parameters based on distinct signatures (e.g. F_ST_, DIND and SFS-statistics) or in at least two parameters based on different features and both in the 1% tails of empirical distributions. Ten genes satisfied these criteria and for all of them analyses were extended to a 100 kb flanking region (50 kb up- and down-stream) to account for the large span of selective sweeps.

As detailed below, we combined multiple tests to identify the most likely selection target (i.e. the advantageous mutation underlying the sweep). Finally, we verified whether these signals could also be detected using other tests based on extended haplotype homozygosity [45], [46].

### Selective sweeps drove the frequency increase of regulatory polymorphisms in APP genes

**Figure 6:**
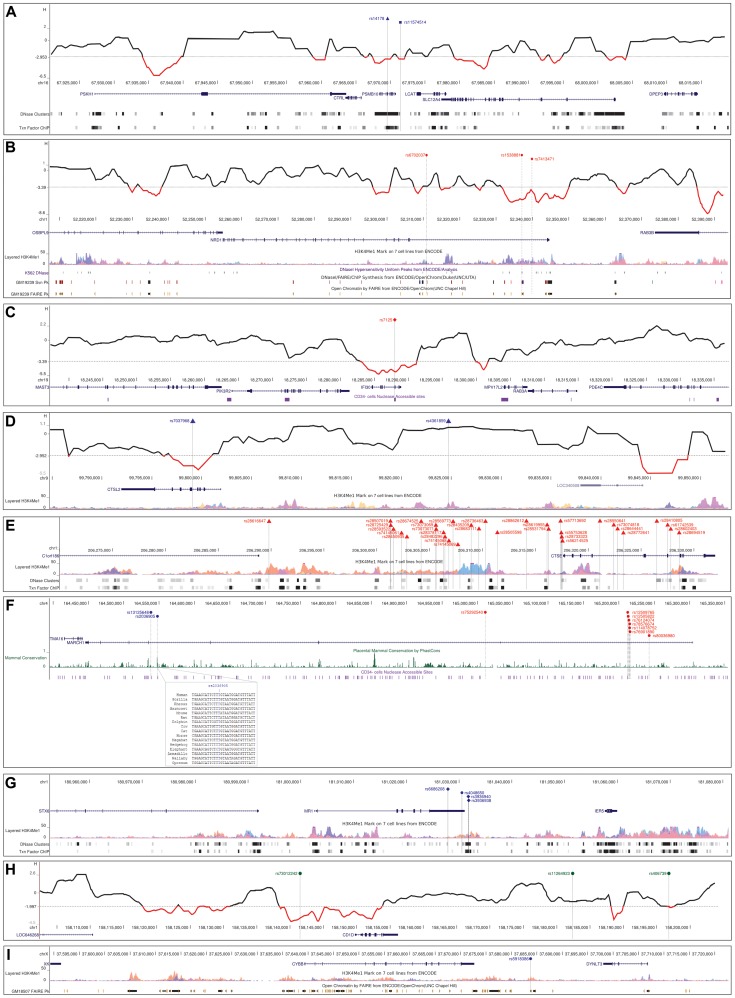
Analysis of selected variants. Location of the most likely selection targets in *PSMB10* (A), *NRD1* (B), *IFI30* (C), *CTSL2* (D), *CTSE* (E), *MARCH1* (F), *MR1* (G), *CD1D* (H), and *CYBB* (I) within the UCSC Genome Browser view. Relevant annotation tracks are shown. For *MARCH1* a short alignment of the highly conserved sequence encompassing rs2036905 is reported. For *PSMB10*, *NRD1*, *IFI30*, *CTSL2*, and *CD1D* a sliding-window analysis of Fay and Wu's H is also shown, as mentioned in the text. The hatched horizontal line represents the 5^th^ percentile (see methods) and significantly negative values are in red. Variants in blue, red and green represent selection targets in CEU, AS, and YRI, respectively. SNP notation is as follows: triangle, F_ST_ outlier; diamond, DIND outlier; dot, both F_ST_ and DIND outlier; square, SNP associated with a disease.

Among genes coding for immunoproteasome-specific subunits, *PSMB10* and *PSME3* showed evidences of selection; nonetheless, variants in *PSME3* might have hitchhiked with a selected allele in a nearby gene, highlighting the need to analyze flanking regions to avoid incorrect inference of selection at a given gene. In fact, *PSME3* showed low diversity and SFS statistics in all populations (Supplementary Figure S5, Supplementary Table S6); in YRI one variant in the gene (rs3785545) had a significant DIND test (Supplementary Figure S6) and represented an outlier in the YRI/CEU F_ST_ distributions (Supplementary Figure S6). Yet, analysis of 5′ and 3′ flanking regions revealed that a SNP (rs61995868) in full linkage disequilibrium (LD) with rs3785545 (r^2^ = 1 in YRI) was an F_ST_ outlier and had a DIND higher than rs3785545. This variant is a nonsynonymous substitution in the nearby *CNTD1* gene and is likely to represent the selection target (Supplementary Figure S7). Conversely, *PSMB10* was subject to directional selection; indeed the gene showed low diversity in CEU and AS (Supplementary Figure S5) and negative Fay and Wu's H in CEU (Supplementary Table S6). One synonymous variant (rs14178) was an outlier in the distribution of YRI/CEU F_ST_ values and in the distribution of DIND-DAF values (Supplementary Figure S6); analysis of 100 kb surrounding the gene revealed no SNP with higher F_ST_ and DIND ranks than rs14178. In CEU the SNP falls in a region of local reduction in Fay and Wu's H, and it is located in the fifth exon of the small *PSMB10* gene (Figure 6A). In this region DNaseI hypersensitive sites and transcription factor binding sites have been mapped by CHIP-seq in several cell lines (Figure 6A). In CEU rs14178 is in full LD (r^2^ = 1) with rs11574514, which is located 1850 bp apart and has been associated with Crohn's disease (CD) in genome-wide association studies [47].

The activity of the proteasome is complemented by cytoplasmic peptidases [2]. One of these, *NRD1*, was found to represent a selection target in Asian populations. The gene showed low diversity (Supplementary Figure S5) and negative SFS-based statistics (Supplementary Table S6); several SNPs were outliers in the YRI/AS F_ST_ distribution and in AS three of these also showed a very high DIND test (Supplementary Figure S6). The three variants had similar DAF (0.94) in AS, and rs1538881 had the highest DIND rank; in an extended region no other variant showed outlier values for DIND and F_ST_. A sliding-window analysis along the region indicated that rs1538881 falls in a valley of Fay and Wu's H calculated on AS chromosomes (Figure 6B). The variant is located at the beginning of the long first intron of the gene, a region where open chromatin signals and H3K4Me1 histone marks have been described in K562 and lymphoblastoid cells (Figure 6B).

Among genes involved in MHC class II presentation, *IFI30* (also known as *GILT*), *CTSE*, and *CTSL2* were found to represent selection targets. Analysis of *IFI30* indicated negative Fay and Wu's H values in AS (Supplementary Table S6) and one outlier SNP (rs7125) in the DIND-DAF distribution for the same population (Supplementary Figure S6). Analysis of the extended region revealed no SNP with higher rank in the DIND test. The variant is synonymous and falls within a nuclease accessible site in CD34- maturing myeloid cells [48] (Figure 6C).

*CTSL2* encodes a cysteine protease also referred to as *CTSV*; analysis of the gene showed a significant negative Fay and Wu's H in CEU (Supplementary Table S6); F_ST_ analysis indicated rs7037968 as an outlier in the CEU/AS distribution (Supplementary Figure S6); analysis of an extended region revealed one single variant with F_ST_ (rs4361859) similar to rs7037968. Sliding window analysis of Fay and Wu's H in CEU indicated that rs7037968 (but not rs4361859) is in a local valley, suggesting that it represents the selection target (Figure 6D). No functional annotation has been described for rs7037968.

As for *CTSE*, encoding cathepsin E, the gene region showed reduced diversity in AS (Supplementary Figure S5) and low Tajima's D and Fay and Wu's D* and F* in this same population (Supplementary Table S6). F_ST_ analysis was performed for all variants in the gene and for genomic flanks, although the region immediately telomeric to *CTSE* is not covered in the human reference sequence, therefore only variants centromeric to the gene were included. Several SNPs were found to be outliers in the YRI/AS F_ST_ distribution (Supplementary Figure S6) and closer inspection revealed that in a number of cases this is due to derived alleles that are fixed or almost fixed in AS, while remain at intermediate frequency in African populations. Most variants cluster in a region upstream *CTSE* or within the transcription unit (Figure 6E), suggesting that a complete/almost complete selective sweep has occurred in AS and targeted *CTSE*; mapping of these variants indicated that many of them fall within potential regulatory regions carrying H3K4Me1 histone marks in different cell types (Figure 6E).

*MARCH1* has also been involved in APP, as it regulates the surface expression of MHC class II molecules [1]. Two variants in the gene (rs2036905 and rs13125648) had an extremely high DIND test in CEU (Supplementary Figure S6) and represented outliers in the YRI/CEU F_ST_ distribution (Supplementary Figure S6). The two variants are located ∼9 kb apart and have similar DAF in CEU (0.61 and 0.66, respectively); interestingly, rs2036905 falls within a sequence that is highly conserved in mammals and affects a position invariant in most species (Figure 6F). In AS, 9 variants with a similar DAF (0.12 to 0.16) had very high DIND test values and represented outliers in the YRI/AS or CEU/AS F_ST_ comparisons or in both (Supplementary Figure S6). Several of these variants are located in a ∼4 kb region in intron 1, and one of them (rs12509765) is within a nuclease accessible site in maturing myeloid cells (CD34- cells) [48] (Figure 6F), suggesting a role in the regulation of *MARCH1* transcription.

Antigen presentation to T cell populations distinct from CD4 and CD8 occurs through specialized molecules encoded by genes that are not located in the MHC. *MR1* showed two variants (rs4048650 and rs6686208) with very high DIND test in CEU and a similar DAF of 0.48 (Supplementary Figure S6); both SNPs are located in the long 3′UTR. rs4048650 also represented an outlier in the YRI/CEU F_ST_ distribution; analysis of an extended region revealed no additional variants showing similarly high DIND and F_ST_ values. rs4048650 is located in the 3′UTR and affects no known microRNA binding site, but it lies in a region showing H3K4Me1 histone marks in lymphoblastoid cell lines (Figure 6G). Consistently, this SNP represents an expression QTL for *MR1* [49]. As for *CD1D*, the gene showed low SFS-based statistics in YRI (Supplementary Table S6). Several variants in the gene and in flanking regions displayed extreme DIND test values in YRI and represented outliers in the YRI/CEU or YRI/AS or in both FST distributions (Supplementary Figure S6). Specifically, one of these variants (rs73012242) is located upstream the transcription start site of *CD1D* and has a DAF of 0.95 in YRI; the remaining variants are positioned downstream the transcription end site and have a DAF ranging from 0.27 to 0.41 (Figure 6H). Sliding window analysis indicated that the 5′ portion of *CD1D* and the upstream region encompassing rs73012242 correspond to a valley of Fay and Wu's H (Figure 6H), suggesting that this SNP represents the selection target at the *CD1D* locus and that the downstream polymorphisms might result from a distinct selective event possibly involving telomeric genes. The derived allele of rs73012242 is fixed in CEU and AS, suggesting that the sweep is complete in these populations. No functional annotation is reported for this variant.

Finally, *CYBB* showed low diversity (Supplementary Figure S5) and negative SFS-based statistics in AS (Supplementary Table S6); in CEU θ_W_ was reduced (Supplementary Figure S5). Analysis of an extended region indicated that one variant (rs5918386) had extremely high DIND test in both CEU and AS and represented an outlier in the YRI/CEU and YRI/AS F_ST_ distributions (Supplementary Figure S6). This variant is located downstream the transcription end site of *CYBB*, in a region where open chromatin and H3K4Me1 histone marks have been described in lymphoblastoid cell lines (Figure 6I). Sliding window analysis was not performed due to the low number of variants segregating in the region.

Finally, we assessed whether the selection signatures we identified above could also be detected using other tests based on extended haplotype homozygosity, namely lnRsb [45] and iHS [46], and if they overlapped with previous positive selection scans. The lnRsb test contrasts extended haplotype homozygosity between two populations and has good power for selective events at high frequency [45], whereas iHS compares the homozygosity decay for haplotypes carrying the ancestral and derived alleles for a given variant in the same population. The test has maximum power for intermediate frequency selective events [46]. As above, an empirical distribution was obtained for lnRsb (CEU/YRI, CEU/AS, and CEU/AS) and iHS values. Six of the selection targets we identified in the analyses above showed very high lnRsb values: rs1538881 in *NRD1* (lnRsb_AS/YRI_: 1.63, rank: 0.951), rs7037968 in *CTSL2* (lnRsb_CEU/AS_: 2.56, rank: 0.988; lnRsb_CEU/YRI_: 2.38, rank: 0.990), most SNPs in *CTSE* and flanking regions (strogest SNP: rs57713692, lnRsb_AS/YRI_: 3.60, rank>0.999), rs2036905 in *MARCH1* (lnRsb_CEU/YRI_: 1.68, rank: 0.950), rs4048650 in *MR1* (lnRsb_CEU/YRI_: 2.30, rank = 0.987), and rs5918386 downstream *CYBB* (lnRsb_CEU/YRI_: 2.62, rank = 0.994) (Supplementary Figure S8). In the case of rs14178, lnRsb was high but not exceptionally so (lnRsb_CEU/YRI_: 1.22, rank = 0.888). As for the iHS test, no variant showed outlier results, the best value being iHS = −1.80 (rank = 0.93) for rs7125 in AS. Nonetheless, it should be noted that most variants we identified have high DAF, thus being difficult to detect through the iHS. Also, the selective event at rs73012242 (upstream *CD1D*) is almost impossible to detect using either lnRsb or iHS as the sweep is at very hight frequency in YRI and likely complete in AS and CEU.

To evaluate the overlap between the signal we detected and those identified in previous scans of positive selection, we retrieved data from 9 genome-wide studies [45], [46], [50]–[56] that applied different approaches. This analysis indicated that large genomic regions covering portions of *MARCH1* had been previously identified in both CEU and AS by Williamson and co-workers [50], who applied a composite likelihood ratio (CLR) model (the *MARCH1* regions have CLR p values <0.01), and by Tang et al. [45], by application of the lnRsb test (Supplementary Figure S9). These latter authors also described a genomic region encompassing *NRD1* as a selection target in AS (Supplementary Figure S9). No overlaps were detected for the remaining genes.

### Balancing selection targeted coding variants in APP genes

Balancing selection is more difficult to detect than positive selection, mainly because its signal (an excess of polymorphism) is often confined to narrow genomic regions [57]. Because the low-coverage 1000 Genomes Pilot Project data are skewed against singletons and low-frequency variants, and because this bias is not homogeneous along the genome, local minor differences might have a comparatively high weight when the selection signal is restricted to relatively small regions. Thus, to obtain unbiased estimates of nucleotide diversity and of the SFS, we Sanger resequenced the putative balancing selection targets in 60 HapMap subjects (20 YRI, 20 CEU and 20 AS).

**Figure 7:**
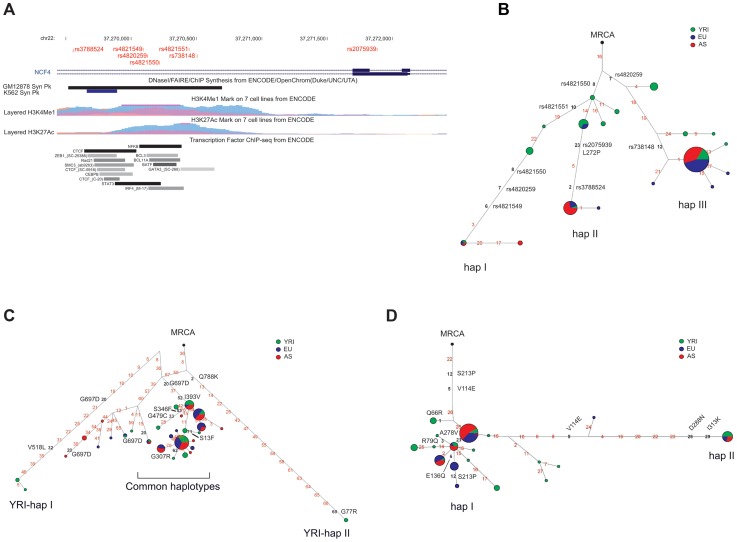
Analysis of *NCF4*, *CD207*, and *TAP1*. (A) Exon-intron structure of the resequenced *NCF4* region with inclusion of a few annotation tracks. The position of SNPs shown in the haplotype network (B) is reported. In the network each node represents a different haplotype, with the size of the circle proportional to frequency. Nucleotide differences among haplotypes are indicated on the branches. The most recent common ancestor (MRCA) is also shown (black circle). The relative position of mutations along a branch is arbitrary. Haplotype phylogenies for *TAP1* (C) and *CD207* (D) were reconstructed through median-joining networks. Nonsynonymous variants are shown, as well as SNPs that fall within potential regulatory elements.

In particular, resequencing was performed for the entire coding sequences of *CD207*, *PSMB9* and *TAP1*. Given the large size of the genes, two sub-regions of 4.6 and 3 kb, respectively were resequenced for *CTSB* and *NCF4* (Figure 7A); these genomic portions were selected because they contain outlier SNPs in the distribution of F_ST_ values (Supplementary Figure S6).

For each analyzed region/gene, nucleotide diversity was assessed by calculating θ_W_ and π; as a control for demographic effects, both indexes were calculated for 5 kb windows deriving from 238 genes resequenced by the NIEHS (National Institute of Environmental Health Sciences) SNP Program. Because under neutral evolution the amount of within-species diversity is predicted to correlate with levels of between-species divergence, we also applied a Maximum-Likelihood-ratio HKA (MLHKA) test [58] to assess whether an excess of polymorphism was observed relative to divergence.

Estimates of nucleotide diversity higher than the 95^th^ percentile were obtained for all genes/regions in at least one population (Table 3, Supplementary Table S7). Nonetheless, a significant excess of nucleotide diversity versus inter-species divergence (as detected by the MLHKA test) was observed only for *CD207* and *TAP1* in YRI, and for *NCF4* in AS (Table 3, Supplementary Table S7). High levels of diversity in human populations that are paralleled by high inter-species diversity (i.e. non-significant MLHKA test) are difficult to interpret and raise the possibility that polymorphisms are not being maintained by selection but result from a high local mutation rate or from relaxation of functional constraints. Thus, we considered candidates of balancing selection only genes/regions that rejected neutrality based on the MLHKA results (in at least one population). For *TAP1*, *CD207*, and *NCF4* we verified whether the neutral model could be rejected by SFS-based statistics through coalescent simulations. Positive values of Tajima's D and of Fu and Li's D* and F* indicate an excess of intermediate frequency variants and are a hallmark of balancing selection, although non-significant SFS statistics may be observed when balancing selection is multiallelic or when balanced haplotypes/alleles are not at intermediate frequency. Significantly high SFS tests were observed for at least one statistic for *TAP1* and *CD207* in YRI, as well as for *NCF4* in AS (Table 3). The values of Tajima's D and of Fu and Li's D* and F* were also compared to the distributions obtained from 5 kb windows deriving from Sanger resequenced NIEHS genes; also these statistics were calculated using the 1000 Genomes Pilot Project data (Supplementary Table S8). Overall, high concordance was observed between coalescent simulation p values and percentile ranks obtained from Sanger sequencing, whereas the 1000 Genomes Project data yielded few values higher than the 95^th^ percentile (Supplementary Table S8), suggesting that Sanger sequencing or high-coverage data may be better suited for the detection of balancing selection.

**Table 3:** Nucleotide diversity and neutrality tests.

Gene	L^a^	Pop^b^	S^c^	θ_w_ d		π^f^		Tajima's D		Fu and Li's D*		Fu and Li's F*		MLHKA	
				value	rank^e^	value	rank^e^	value	*p* g	value	*p* g	value	*p* g	K^h^	*p* i
***CD207***	4.7	YRI	40	19.90	**0.98**	20.13	**0.98**	0.04	0.10	0.51	**0.045**	0.41	**0.042**	2.53	**0.014**
		CEU	27	13.43	**0.96**	12.06	0.87	−0.35	0.25	0.68	0.13	0.39	0.26	2.22	0.066
		AS	20	9.95	0.90	9.13	0.82	−0.27	0.25	1.32	**0.013**	0.93	0.099	1.77	0.39
***NCF4***	3.0	YRI	20	15.60	0.94	15.33	0.94	−0.056	0.24	−0.75	0.38	−0.61	0.49	1.97	0.13
		CEU	17	13.26	**0.95**	11.31	0.88	−0.48	0.31	−1.14	0.18	−1.08	0.19	2.31	0.071
		AS	16	12.48	**0.96**	17.88	**0.97**	1.39	0.098	1.58	**0.007**	1.79	**0.016**	2.27	**0.039**
***TAP1***	7.2	YRI	53	17.17	**0.97**	10.98	0.79	−1.29	0.098	1.32	**0.0017**	0.49	0.057	2.92	**0.0065**
		CEU	24	7.77	0.72	6.14	0.60	−0.71	0.19	−0.66	0.29	−0.80	0.23	1.66	0.73
		AS	23	7.45	0.77	7.41	0.40	−0.018	0.41	−0.45	0.32	−0.36	0.33	1.75	0.77

a length of analyzed resequenced region (in kb);

b population;

c number of segregating sites;

d θ_w_ estimation per site (×10^−4^);

e percentile rank relative to a distribution of 238 5 kb windows from NIEHS genes;

f π estimation per site (×10^−4^);

g *p* value calculated by coalescent simulations;

h selection parameter (k>1 indicates an excess of polymorphism compared to divergence; k<1 indicates the opposite situation);

i *p* values obtained by applying a calibrated population genetics model, as described in the text.

To further extend these analyses, haplotype phylogenies were reconstructed for *NCF4*, *TAP1*, and *CD207*. The haplotype phylogeny for the resequenced NCF4 region showed 3 main haplotype groups (hapI–III, Figure 7B) with an estimated time to the most recent common ancestor (TMRCA) ranging from 840,000 to 1,790,000 years (Supplementary Table S9, Supplementary Figure S10). One of them (hap I) has low frequency in all populations and carries putative regulatory variants (Figure 7A–B). Hap II carries the derived allele of rs3788524, which is an outlier in the YRI/AS F_ST_ distribution (Supplementary Figure S6); in AS and CEU this variant is in strong LD with L272P (rs2075939), which also defines HapII. The derived allele of a putative regulatory variant (rs738148) defines hapIII (Figure 7A–B). Overall, these data support a scenario of multiallelic balancing selection at the *NCF4* gene, with both missense and regulatory variants being maintained in human populations.

In the case of *TAP1*, the haplotype network showed a complex scenario and revealed a few recurrent mutations, possibly originating from recombination or gene conversion. One major cluster of haplotypes is evident, and all these chromosomes carry the derived alleles at aminoacid residues 393 and 697 (393I and 697D). Two distantly related haplotypes are observed in YRI (YRI-hapI and YRI-hapII, Figure 7C) and both carry at least one distinctive nonsynonymous variant (V518L and G77R plus Q788K, respectively). The presence of highly differentiated haplotypes with restricted geographic distribution might be suggestive of ancient population structure [59]; nonetheless, calculation of the TMRCA for the haplotype phylogeny yielded estimates ranging from 1,670,000 to 660,000 years (Supplementary Table S9, Supplementary Figure S10),which are not consistent with population structure in Africa. Although some variants that affect putative gene transcription regulatory elements are also located on the branches of the haplotype genealogy, the balancing (or diversifying) selection targets are likely to be accounted for by aminoacid substitutions.

Finally, the haplotype network of *CD207* was reconstructed using variants located in a sub-region of relatively tight linkage disequilibrium (covering the whole transcription unit with the exclusion of exon 1 and intron 1); nonetheless, some recurrent mutations were evident (Figure 7D). The two major haplogroups carry different alleles at two polymorphisms that affect residues in the CRD: N288D, which was shown to affect binding to mannose [60], and K313I, where the lysine residue forms the sulfated glycan recognition interface [61]. Within the more common haplotype cluster, other missense variants are observed, including A278V, which does not influence sugar binding or protein stability [60]. Few CEU chromosomes are differentiated at the S213P variant (Figure 7D); reconstruction of ancestral state at this site is difficult as different primates carry distinct residues, in line with the fact that this position was found to be positively selected in mammals (Figure 3). Overall, these data suggest that in humans balancing selection targeted two nonsynonymous variants -K313I and N288D- resulting in two major langerin forms (288N-313K and 288D-313I), that segregate in human populations and are likely different in their sugar binding specificity.

CD207/langerin can internalize HIV-1 to Birbeck granules where it is degraded [62]. Thus, we explored the possibility that the selected functional variants in the CRD domain affect the susceptibility to sexually-transmitted HIV-1 infection. To this aim, we genotyped rs13383830 (N288D) in a cohort of 87 Italian heterosexual HIV-exposed seronegative (HESN) individuals who have a history of unprotected sex with their seropositive partners [63] and in 436 randomly selected Italian subjects (controls). The variant significantly deviated from Hardy-Weinberg equilibrium (HWE) with an excess of homozygotes in HESN alone (Table 4). This observation may be explained by the underlying genetic model (i.e. protection from HIV-1) or by spurious effects; application of a goodness-of-fit test [64] indicated that a recessive model with only genetic effects adequately explains HWE deviation in HESN. Comparison of allele frequencies in the HESN and control samples indicated no significant difference for rs13383830. Conversely, the genotype distribution of the SNP was significantly different in the two cohorts, with 288D/288D homozygotes being much more common in HESN than in controls (permutation p = 0.015 and 0.023 for a genotypic and a recessive model, respectively, Table 4). Thus, homozygosity for the 288D allele may be a factor in determining protection from sexually-transmitted HIV-1 infection.

**Table 4:** Genotype counts, HWE proportions and association analysis for rs13383830.

SNP ID	Phenotype	Genotype counts	Genotype counts (recessive model)	*p* a (HWE)	*p* b (genotype)	*p* b (recessive)
**rs13383830 (N288D)**	**HESN**	3/12/72	3/84	0.04	0.015	0.023
	**CTR**	2/59/375	2/434	>0.99		

a HWE deviation p value.

b *p* value obtained from 10,000 phenotype-label swapping permutations.

## Discussion

Adaptive evolution acts at the level of genetic variants that determine advantageous phenotypic traits. Selection signatures can therefore be exploited to detect genomic regions/positions underlying phenotypic diversity and adaptation. This has recently been demonstrated within a host-pathogen arms race scenario whereby an evolutionary-guided approach was used to identify a protein loop in MX1 (myxovirus resistance 1) that determines antiviral activity [65]. Similarly, it has been known for years that natural selection has specifically acted on the peptide-binding cleft of antigen presenting molecules [4]. Because the repertoire of peptides that is available for presentation is generated by APP gene products, we performed an evolutionary analysis of these loci.

Evolutionary analysis at the inter-specific level indicated that 11 genes have been targeted by diversifying positive selection; this represents a substantial fraction (24%) of analyzed genes, despite our application of a conservative approach. Moreover, an analysis of positive selection in the human species identified positively selected codons at four additional genes. Although large-scale analyses had previously identified immune response loci as preferential targets of positive selection in mammals [66], [67], those studies had limited power due to the inclusion of a small number of species. Thus, in Kosiol et al. [66] the percentage of positively selected genes among those involved in APP only amounted to 14%.

Likewise, we identified several genes targeted by natural selection during the more recent history of human populations. Integration of different tests for selection was recently shown to be a powerful tool to identify and finely map positive selection targets [68]; the approach we applied herein differs in a number of ways from that proposed by Grossman and co-workers [51], [68]. We did not apply the integrated haplotype score (iHS) or its derivatives, but rather relied on the DIND test, which was proven to be more powerful than iHS in most ranges of selected allele frequency [42]. We used the normalized H statistic (as it has higher power than the original non-normalized Fay and Wu's H [39]) rather than the ΔDAF test [51], [68], and we included SFS-based statistics. Thus, due to the different power of distinct tests, none of the variants described herein was identified in previous scans for positive selection. At the gene level, analysis of genome-wide scans of positive selection indicated that regions encompassing *MARCH1* and *NRD1* had previously been described as positive selection targets [45], [50], whereas no overlap was detected for the remaining genes. Low concordance of positive selection signals among studies has been previously noted (for a recent review [69]). However, most previous positive selection scans have been performed using SNP genotype data (however dense in some cases) whereas we used resequencing data (although low-coverage), which are expected to increase the power to detect selection [70]. Indeed, even tests based on extended haplotype homozygosity, that are less sensitive to the ascertainment bias, have increased power when the actual selection target is included in the analysis [46]. One extreme example of this is accounted for by *CTSE*, where no SNP mapped in HapMap releases predating 2008 and which is still poorly covered by HapMap data.

Several reports have indicated that genes involved in immune response may be preferential targets of both positive [45], [46], [51], [56] and balancing [71] selection in humans, with some immune-response pathways possibly being particularly enriched in selection signals. Tang et al. [45] found an over-representation of genes coding for cytokines (IL-1 receptor agonists in particular) among their top signals; likewise, other authors indicated an enrichment for complement-mediated and class I MHC-related immune response genes [46], [51]. Beside genome-wide scans, studies that focused on specific families of immune response loci often revealed a high proportion of selected genes; these include, for example, type III interferon genes [72], genes coding for T-cell regulatory molecules [73], and NOD-like receptors [74]. These observations clearly reflect the extremely important role of immune response for survival in the face of infections. Nonetheless, analyses herein also indicated that for some components of the APP pathway (e.g. immunoproteasome subunits, chaperones, several lysosomal proteases) negative selection likely represented the major evolutionary force. Conversely, genes that code for APP components that, at different levels, directly interact with the antigens to be presented (e.g. *CD1D*, *CD207*, *TAP1*, *ERAP2*, and *CYBB*) have been constantly targeted by positive or balancing selection, as determined by both inter- and intra-species analyses.

Besides providing a general picture of the evolutionary forces acting on the APP pathway, our aim was to describe in detail the specific sites and variants targeted by natural selection so that this information can be exploited to prioritize functional characterization in follow-up analyses. We defined positively selected sites in mammals by the combined use of two methods, BEB and MEME; this choice was taken to limit the number of false positive results, although we most likely underestimated the number of selected sites. In fact, MEME was developed to detect both episodic and pervasive positive selection [9], whereas sites evolving under episodic selection are likely to be missed by BEB. Thus, the combination of the two methods is expected to result in the confident identification of sites evolving under pervasive diversifying selection only.

Nonetheless, several sites evolving adaptively were identified and they are expected to define positions and protein regions that affect functional properties. As an example, our data indicate that a threonine residue (322T) that functions as a trafficking signal in the cytoplasmic region of CD1D [13] is present in primates only and represents a selected site, suggesting that different motifs evolved in distinct mammalian species to modulate CD1D expression at the plasma membrane. Indeed, differences in intracellular trafficking between mouse and human CD1D molecules have been reported [75]. Interestingly, it has been proposed that the 322T signal is exploited by HSV-1 to down-modulate the surface expression of CD1D molecules as an evasion strategy [13]. Thus, the cytoplasmic tail and the transmembrane region of CD1D might have evolved under virus-driven selective pressure. Indeed, different pathogens, including HSV-1, HPV, HIV-1, VSV, and KSHV, interfere with CD1D expression and recycling [75], although the specific contact interfaces between viral products and CD1D molecules are unknown. Adaptive evolution was also evident in the extracellular domains of CD1D; sites positively selected in mammals are spatially clustered and flank the TcR interaction surface and the lipid binding pocket, suggesting that they may exert indirect effects on binding specificity, especially in light of the broad array of lipid molecules presented by CD1D [75]. Similarly, a human-specific positively selected site at the α2/α3 domain interface might modulate CD1D activity by altering the flexibility or relative positioning of the extracellular domains.

Different viral species are known to encode products that counteract specific components of the APP pathway other than CD1D. This represents a strategy to evade the host immune system by hampering the presentation of immunogenic epitopes. Specifically, several viral proteins target the PLC by binding TAP or tapasin [5]. Viral inhibition of the PLC is suggested to be of pivotal importance for efficient infection; for example different herpesviruses encode distinct TAP inhibitors, which are unrelated in genome location, structure, and mechanism of action, suggesting convergent evolution [76]. This indicates that some of the positively selected sites we identified in TAP1 and tapasin (TAPBP) might have evolved to avoid targeting by viral products. One of these is the US3 immunomodulator encoded by HCMV; this protein directly binds the tapasin ER luminal domain, but has no effect on the formation of the TAP-tapasin complex [5]. US3 might interfere with recruitment of ERp57 by tapasin [5], suggesting that the tapasin 225N residue -located at the ERp57 binding interface - might be involved in this process.

Three of the positively selected sites in TAP1 are located in the channel forming region and one of them (516Q) maps to a transmembrane domain that directly interacts with peptides. Because TAP is known to select peptides for transportation in a species-specific manner [77], it would be interesting to evaluate the effect of the identified residues on TAP binding affinity and transportation preference, as well as on the sensitivity to viral inhibitors. TAP contributes to the shaping of the overall repertoire available for MHC presentation. On the one hand this property *per se* represents a possible target for host-pathogen arms races, as decreasing transport of specific peptides would translate in reduced presentation. On the other hand, it has been noticed that in human, mouse, and rat, the specificity of TAP transportation correlates with the predominant peptide binding profiles of the corresponding MHC class I molecules, suggesting co-evolution [77].

Co-evolution with MHC class I molecules might also be driving aminoacid replacements at BLMH and tapasin. Indeed, the N-terminal domain of tapasin, where one of the selected residues (67S) is located, was shown to facilitate MHC-peptide complex folding depending on the identity of both the peptide and of the HLA I heavy chain. As for BLMH, experiments in human cells indicated that its depletion affects peptide loading and MHC class I surface expression in a *HLA* class I allele-dependent manner [78]. *BLMH* is highly conserved from yeast to mammals, suggesting strong constraints [27]. As a consequence, selection might have acted at the level of aminoacid residues that modulate protein abundance at the post-translational level, as suggested by their location. Likewise, natural selection might have acted at the *ERAP2* locus to modulate protein stability and, consequently, abundance. Although the observation that protein-destabilizing variants have been favored during evolution might seem counter-intuitive, it should be noted that an *ERAP2* haplotype that results in a truncated (and degraded) protein product is maintained by balancing selection in human populations [33], [44]. Also, some rodent species, including mice and rats, lack a functional *ERAP2* gene, suggesting that loss or decreased abundance of ERAP2 protein might confer some advantage, possibly related to selective antigen trimming. We also detected human-specific selective events at *ERAP1*. One of the two variants we identified had previously been shown to represent a balancing selection target in human populations [33]. The variant affects enzymatic properties [79] and associates with the susceptibility to different autoimmune diseases, often in interaction with *HLA* allelic status [80].

Analysis of *CYBB* and *CD207* also provides remarkable examples of the action of different selective forces on the very same gene region, as both highly variable and strongly constrained positions are observed in close proximity at these loci. Indeed, most missense substitutions that cause mendelian immunologic defects involve aminoacid positions that are conserved in all mammals, indicating that negative selection at these sites prevents aminoacid replacements affecting host resistance to pathogens. The pattern of positive selection at *CYBB* indicates that the two long loops protruding in the extracellular space or in the phagosome lumen are strongly targeted by diversifying selection. These protein regions are expected to be mostly exposed to a direct interaction with pathogen components, suggesting that they have evolved to avoid inhibition by bacterial/fungal products, a possibility that awaits experimental validation. In addition to its role in cross-presentation, the NADPH oxidase complex directly participates in the killing of pathogenic microbes through the production of superoxide and other oxidants in neutrophils. This activity is also required to activate cathepsin G and other proteases that, in turn, kill and digest engulfed pathogens [81]. Most positively selected sites we identified in *CTSG* are likely to modulate substrate specificity as they rim the binding pockets. Likewise, the site targeted by positive selection in simians is located at the edge of the substrate binding pocket on an exposed loop that also carries 177G (positively selected in the whole phylogeny); this loop has previously been shown to confer substrate specificity to other serine proteases [25], [82]. Interestingly, a site subject to diversifying selection (106Q) is adjacent to a position (104T) that, if replaced with the equivalent aminoacid in elastase (T104N), confers to CTSG the ability to cleave *Shigella* virulence factors [82] (Figure 3B). Thus, the selective pressure acting on both *CYBB* and *CTSG* might be related to their direct antimicrobial role in addition to participation in APP. Finally, analysis of human-specific positively selected sites in *LGMN*, which also encodes a lysosomal protease, indicated that one of them maps to the α cleavage site of the activation peptide. Although the identity of the protease(s) responsible for cleavage is presently unknown, the multistep activation of LGMN is thought to have a regulatory significance and is modulated by the maturation status of dendritic cells, possibly via acidification of the endosome/lysosome compartments [83].

Results herein also indicate a continuum in selective pressure acting on different timescales and targeting the coding sequences of *TAP1* and *CD207*, as aminoacid-replacement variants are likely to represent the selection targets in human populations. In both cases balancing selection signatures were detected in African populations only. Because we accounted for demography events both in coalescent simulations and by the empirical comparison with genes resequenced in the same populations, the signatures we detect are unlikely to represent demographic effects, but instead indicate stronger selective pressure in Africa. Interestingly, one of the putative balancing selection targets in *TAP1*, the V518L variant, is located in the peptide binding domain, close to a positively selected site (516Q), and defines a minor haplotype in YRI; this variant might affect the affinity of TAP1 for one or more antigenic peptides. Likewise, in the case of *CD207* one positively selected site (289A) immediately flanks a human polymorphic position representing a balancing selection target (N288D) with known effect on sugar binding [60]. The second site subject to diversifying selection (213P) is polymorphic in humans (P213S), although its positioning on the haplotype network does not suggest that it is a major target of balancing selection in humans. Indeed, the two major haplotype clades of *CD207* carry, in addition to N288D, a second variant, K313I, that also affects langerin binding to glycan substrates [61]. This indicates that balancing selection has maintained two alternative langerin forms that differ in binding specificity and may recognize distinct microbial glycan structures, ultimately affecting the susceptibility to specific infections. We show that homozygosity for the 288D-313I langerin haplotype may be associated with protection against sexually transmitted HIV-1 infection. The HIV-1 gp120 protein, which is bound by CD207, is heavily glycosylated with both oligomannose and complex N-glycans [84]; the 288D allele displays reduced binding to mannose-containing structures [60], but may confer increased affinity for more complex sugars, as suggested by the broad specificity of langerin. Overall, although the recessive effect of the rare haplotype is consistent with the trimeric nature of langerin, and its frequency differs in HESN and controls (3.45% and 0.46%, respectively), the association results should be regarded as preliminary and treated with caution due to the small sample size and the low frequency of the putative protective haplotype. Thus, replication in an independent cohort and functional analyses on the role of the 288D allele in HIV-1 recognition and internalization will be needed.

One nonsynonymous polymorphism (L272P) in *NCF4*, encoding a cytosolic regulatory component of the NADPH oxidase complex, was also identified as a possible balancing selection target in human populations. This SNP is located in an intron of the gene that may be retained in the transcript as a result of alternative splicing. Nonetheless, the selection target might also be accounted for by variants with a regulatory function on *NCF4* expression. Indeed SNPs located on the branches of the haplotype genealogy fall within Chip-seq mapped binding sites for transcription factors including STAT3, which is regulated by RAC1 [85], a modulators of NAPH oxidase activity [86], and NFKB, a central transcriptional regulator in myeloid cells. Similarly, we found all adaptive variants subject to directional selection to represent likely modulators of gene expression levels.

As recently suggested [68], the use of large-scale low coverage data, while posing challenges due to the biased SFS, may allow identification of the causal variant underlying the selective event. This represents a valuable advantage by providing a list of targets that may be directly tested in functional analyses. Moreover, integration of selection signals with extensive functional annotations generated by the ENCODE project and by eQTL studies further increases the possibility to underscore adaptive alleles. Our analysis indicated that two of the selected variants (in *IFI30* and *MARCH1*) are located within nuclease accessible sites in maturing myeloid cells, suggesting they affect transcription regulatory elements activated during cell differentiation [48] and the selected variant in *MR1* represents an eQTL. Likewise, selected variants in or close to *NRD1* and *CYBB* fall within open chromatin regions in lymphoblastoid cell lines, and the synonymous variant in *PSMB10* maps to DNAse I sensitive sites in different cell types and to transcription factor binding sites. Interestingly, this variant is in full LD in CEU with a risk SNP for Crohn's disease [47], again supporting the view that adaptive events underlie phenotypic variability. In general, most of the positive selection events we described occur at positions with a likely role controlling gene expression. Grossman and co-workers [68] finely mapped causal variants in 412 candidate selected regions and determined the large majority of these may modulate transcription levels. Likewise, Vernot et al. [87] performed a genome-wide analysis of DNase I hypersensitive regions and indicated that these harbor a number of variants targeted by positive selection in human populations. Thus, our data are in agreement with previous findings and help substantiate the view that regulatory variation represents a major target for adaptive evolution in humans.

## Materials and Methods

### Gene selection

The initial list of genes to be included in the study was obtained from Gene Ontology (GO). Specifically, we queried GO for all the all human genes (n = 180) associated with the following GO terms (and children): GO:0019884 (antigen processing and presentation of exogenous antigen), GO:0019883 (antigen processing and presentation of endogenous antigen), GO:0002474 (antigen processing and presentation of peptide antigen via MHC class I), GO:0002495 (antigen processing and presentation of peptide antigen via MHC class II), GO:0002428 (antigen processing and presentation of peptide antigen via MHC class Ib). From this initial list we removed *HLA* class I (n = 7) and class II genes (n = 15), as they have been the topic of intense investigation, as well as immunoglobulin receptors (n = 3) and integrins (n = 2), as they are not directly involved in the process that leads to antigen processing and presentation (APP). We also pruned genes that, although participating in APP, play non-specific roles including components of the constitutive proteasome (n = 34), general ubiquitination factors (n = 4), molecules involved in the formation and transport of clathrin-coated vescicles (n = 19), proteins involved in vesicle trafficking across different cellular compartments (n = 14), dynamins and dyneins (n = 11), dynactins (n = 6), and kinesins (n = 19). Two ubiquitine-ribosomal protein gene fusions were discarded as well, as their function is poorly understood. Finally, *HFE*, encoding a nonclassical MHC class Ib molecule, was discarded because this gene is believed to have no antigen-presentation function [88]. Thus, we concentrated our efforts on a list of 43 genes, which are considered to be central components of the APP pathway. Notably, *THOP1* and *NRD1* were also included in the final group of genes given their recently established role in antigen processing [2]; this lead to a final list of 45 genes (Supplementary Table S1).

### Evolutionary analysis in mammals

Mammalian sequences for APP genes were retrieved from the Ensembl database. Mammalian orthologs of human APP genes were included only if they represented 1-to-1 orthologs as reported in the EnsemblCompara GeneTrees [89]. As mentioned in the text only primate sequences were included for *CTSL1* and *CTSL2* (Supplementary Table S2).

DNA alignments were performed using the RevTrans 2.0 utility [90], which uses the protein sequence alignment as a scaffold for constructing the corresponding DNA multiple alignment. This latter was checked and edited by hand to remove alignment uncertainties. Trees were generated by maximum-likelihood using the program DnaML (PHYLIP Package). To detect selection, NSsite models that allow (M2a, M8,) or disallow (M1a, M7) sites to evolve with dN/dS>1 were fitted to the data two models of equilibrium codon frequencies: the F3x4 model (codon frequencies estimated from the nucleotide frequencies in the data at each codon site) and the F61 model (frequencies of each of the 61 non-stop codons estimated from the data). Results for the two codon frequency models are reported in Supplementary Tables S3 and S4. Whenever maximum-likelihood trees showed differences (always minor) from the accepted mammalian phylogeny, analyses were repeated using the accepted tree, and the same results were obtained in all cases. Sites under selection with the M8 model were identified using Bayes Empirical Bayes (BEB) analysis with a significance cutoff of 0.90 [8], [91].

In order to identify specific branches with a proportion of sites evolving with ω>1, we used BS-REL [10]. Branches identified using this approach were cross-validated with the branch-site likelihood ratio tests from PAML (the so-called modified model A and model MA1, “test 2”) [11]. A false discovery rate correction was applied to account for multiple hypothesis testing (i.e. we corrected for the number of tested lineages), as suggested [12]. BEB analysis from MA (with a cut-off of 0.90) was used to identify sites that evolve under positive selection on specific lineages. Ancestral site reconstruction for positions 416 and 420 in ERAP2 was obtained through the DataMonkey sever by ASR utility, which implements three different methods. GARD [92], MEME [9], SLAC [93], and BS-REL [10] analyses were performed through the DataMonkey server [94] (http://www.datamonkey.org).

### In silico analysis of protein stability

Intra-protein interaction calculations were performed using PIC (Protein Interactions Calculator) [95]. Stability analysis was carried out using three different methods. FoldX 3.0 [96] and PoPMuSiC (web-server version) [97], were used on the chain A of the X-ray structure of ERAP2 (PDB code: 3SE6). I-Mutant 2.0 [98] was used on the corresponding protein sequence retrieved from UniprotKB (Q6P179). In FoldX and I-Mutant the ΔΔG values are calculated as follows: *ΔΔG = ΔG_mutant_−ΔG_wild-type_*. In FoldX and I-Mutant ΔΔG values >0 kcal/mol indicate mutations that decrease protein stability, whereas in PoPMuSiC ΔΔG values >0 kcal/mol are mark of mutation increasing protein stability. Therefore, PoPMuSiC ΔΔG values were multiplied by −1 to obtain homogeneous results.

In the analysis carried out with FoldX 3D, the three-dimensional structure of the protein was repaired using the <RepairPDB> command. Mutations were introduced using the <BuildModel> command with <numberOfRuns> set to 5 and <VdWdesign> set to 0. Temperature (298K), ionic strength (0.05 M) and pH (7) were set to default values and the force-field predicted the water molecules on the protein surface. Residues His370, His374, Glu393 and Tyr455, which coordinates the zinc ion, were kept fixed during reparation and mutation procedures.

### HapMap DNA samples and sequencing

Human genomic DNA from HapMap subjects (20 Yoruba, YRI, 20 European, CEU, and 20 Asians, AS) was obtained from the Coriell Institute for Medical Research. All analysed regions were PCR amplified and directly sequenced. PCR products were treated with ExoSAP-IT (USB Corporation Cleveland Ohio, USA), directly sequenced on both strands with a Big Dye Terminator sequencing Kit (v3.1 Applied Biosystems) and run on an Applied Biosystems ABI 3130 XL Genetic Analyzer (Applied Biosystems). Sequences were assembled using AutoAssembler version 1.4.0 (Applied Biosystems), and inspected manually by two distinct operators. All primers sequences are available in Supplementary Table S10.

### Population genetics-phylogenetics analysis

Data from the Pilot 1 phase of the 1000 Genomes Project were retrieved from the dedicated website [31]. SNP genotypes were organized in a MySQL database. Coding sequence information was obtained for the 45 APP genes. Accessibility of gene region by paired-end next-generation sequencing was evaluated using the “1000 Genomes Project Phase 1 Paired-end Accessible Regions - Pilot Criteria” UCSC track.

To analyze the DFE for APP genes we used gammaMap [30]. We assumed θ (neutral mutation rate per site), k (transitions/transversions ratio), and T (branch length) to vary among genes following log-normal distributions. For each gene we set the neutral frequencies of non-STOP codons (1/61) and the probability that adjacent codons share the same selection coefficient (p = 0.02). For selection coefficients we considered a uniform Dirichlet distribution with the same prior weight (0.1) for each selection class. For each gene we run 100,000 iterations with thinning interval of 10 iterations.

### Population genetic analyses

A set of programs was developed to retrieve genotypes from the 100 Genomes Pilot Project MySQL database and to analyse them according to selected regions/populations. These programs were developed in C++ using the GeCo++ [99] and the libsequence [100] libraries. Genotype information was obtained for the 45 APP genes. In order to obtain a control set of ∼1,000 genes to use as a reference set, we initially selected 1,200 genes by random sampling of those included in the RefSeq list. For these genes we retrieved orthologous regions in the chimpanzee, orangutan or macaque genomes (outgroups) using the LiftOver tool; genes showing less than 80% human-outgroup aligning bases were discarded. This originated a final set of 987 genes, hereafter referred to as control set. These data were used to calculate θ_W_ [36], π [37], as well as Tajima's D [38], Fu and Li's D* and F* [40], and normalized Fay and Wu's H [39], [101] over each entire gene region.

Data from the control gene set were used to calculate empirical distributions of these parameters, as specified in the text.

Normalized Fay and Wu's H was also calculated in 5 kb sliding windows moving with a step of 500 bp. Sliding window analyses have an inherent multiple testing problem that is difficult to correct because of the non-independence of windows. In order to partially account for this limitation, we applied the same procedure to the control gene set, and the distribution of normalized Fay and Wu's H was obtained for the corresponding windows. This allowed calculation of the 5^th^ percentile and visualization of regions below this threshold.

F_ST_ [41] and the DIND test [42] were calculated for all SNPs mapping to the control and APP gene sets. Because F_ST_ values are not independent from allele frequencies, we binned variants based on their MAF (50 classes) and calculated the 95^th^ and 99^th^ percentiles for each MAF class. As for the DIND test, it was originally developed for application to Sanger or high coverage sequencing data [42], so that statistical significance can be inferred through coalescent simulations. This is not the case for the 1000 Genomes Project data; thus, we calculated statistical significance by obtaining an empirical distribution of DIND-DAF value pairs for variants located within control genes. Specifically, DIND values were calculated for all SNPs using a constant number of 40 flanking variants (20 up- and down-stream). The distributions of DIND-DAF pairs for YRI, CEU and AS was binned in DAF intervals (100 classes) and for each class the 95^th^ and 99^th^ percentiles were calculated. As suggested previously [42], for values of iπ_D_ = 0 we set the DIND value to the maximum obtained over the whole dataset plus 20. Due to the nature of low-coverage data, for low DAF values most iπ_D_ resulted equal to 0 (i.e. the 95^th^ percentile could not be calculated); thus, we did not calculated DIND in these ranges and we consequently cannot detect selection acting on low frequency derived alleles.

The lnRsb and iHS tests were calculated as previously described [45], [46] using the rehh R package [102]. Specifically, lnRsb and iHS were calculated for all tested SNPs using information from 200 kb flanking regions (100 kb 5′ and 3′). To obtain empirical distributions, we randomly selected 100 genic SNPs and calculated lnRsb and iHS values for all SNPs in their 200 kb flanks. Data obtained from these randomly selected variants were alos used to calculate the median and standard deviation for lnRsb' normalization [45].

As mentioned in the text, an approach based on coalescent simulations was applied with Sanger sequencing data. In particular, calibrated coalescent simulations were performed using the cosi package [103] and its best-fit parameters for YRI, CEU, and AS populations with 10,000 iterations. Demographic parameters for YRI, CEU and AS implemented in cosi are described in [103]. Simulations were conditioned on mutation and recombination rates. Estimates of the population recombination rate parameter ρ were obtained from resequencing data with the use of the Web application MAXDIP [104] and converted to cM/Mb.

For Sanger-resequenced regions the percentile ranks of θ_W_ and π were obtained from the distribution of the same parameters calculated for 5 Kb windows deriving from 238 human genes resequenced by NIEHS (National Institute of Environmental Health Sciences) SNPs Program, as previously described [105]. The maximum-likelihood-ratio HKA test was performed using the MLHKA software [58], as previously proposed [105].

### Haplotype analysis and TMRCA calculation

Haplotypes were inferred from Sanger resequencing data using PHASE version 2.1 [106], [107]. Median-joining networks to infer haplotype genealogy were constructed using NETWORK 4.5 [108]. Estimates of the time to the most common ancestor (TMRCA) was obtained using different methods: i) a phylogeny based approach implemented in NETWORK 4.5 using a mutation rate based on the number of fixed differences between chimpanzee and humans [108]; ii) GENETREE, which is based on a maximum-likelihood coalescent method [109], [110] assuming an infinite-site model without recombination; haplotypes and sites that violate these assumptions were removed; iii) a previously described method [111] that calculates the average pairwise difference between all chromosomes and the MRCA: this value was converted into years on the basis of mutation rate retrieved as above. The SD for this estimate was calculated as previously described [112].

We based calculations on the assumption that the divergence between human and chimpanzee occurred 6 MY ago [113] and that the generation time is 25 years.

### Human subjects, genotyping and association analysis

Inclusion criteria for HESN were a history of multiple unprotected sexual episodes for more than 4 years at the time of the enrolment, with at least 3 episodes of at-risk intercourse within 4 months prior to study entry and an average of 30 (range, 18 to >100) reported unprotected sexual contacts per year. These HESN subjects are part of a well characterized cohort of serodiscordant heterosexual couples that has been followed since 1997 (reviewed in [63]).

No HESN was homozygous for the *CCR5Δ32* variant, which confers resistance to R5 HIV-1 strains [114]. As for controls, 436 Italian donors were also included in the study, irrespective of their HIV infection status. The study was reviewed and approved by the institutional review board of the S. M. Annunziata Hospital, Florence. Written informed consent was obtained from all subjects.

HWE deviation was analysed as suggested by Wittke-Thompson and co-workers [64]. The equations are parametrized in q (susceptibility allele frequency), α (risk in non-susceptible homozygotes), β (heterozygote relative risk), γ (homozygote relative risk) and K_p_ (trait prevalence in the general population). We obtained ML estimates for these parameters minimizing the goodness-of-fit test statistic (as reported in [64]) using the BFGS method. Using an estimate of K_p_ the procedure was repeated with a general model estimating q, β and γ, and for constrained specific models, estimating q and gamma (dominant: β = γ; recessive: β = 1, γ>1; additive: β = (γ+1)/2, γ>1; multiplicative: β = sqrt(γ), γ>1). Given the different number of parameters in the general model, the Akaike Information Criteria (AIC) was used for the best fit model selection. A p value was then calculated for the minimal value of the test statistic using a χ_2_ distribution with 1 or 2 df for the general and constrained models respectively. Using a K_p_ (prevalence of HESN phenotype in the general population) of 0.20 [115], [116], the best model fitting the genotypic proportions in HESN and controls was a recessive model with q (susceptibility allele frequency) = 0.079, α (risk in non-susceptible homozygotes) = 0.20, β (heterozygote relative risk) = 1, and γ (homozygote relative risk) = 3.23. For this model, the goodness-of-fit test was not significant (χ_2_ = 1.81, p = 0.40, df = 2), indicating that a recessive model with only genetic effects adequately explains HWE deviation. We performed the same analysis using a range of K_p_ (from 0.10 to 0.30) and similar results were obtained (not shown).

Association p values for the genotypic and recessive models were calculated using PLINK [117] by performing 10,000 phenotype-label swapping permutations.

## Supporting Information

Figure S1 Work-flow and main results for the inter-species analysis. Genes that were defined as targets of positive selection are shown in red.
(PDF)

Figure S2 Branch-site analysis of positive selection for *CYBB*. Branch lengths are scaled to the expected number of substitutions per nucleotide, and branch colors indicate the strength of selection (dN/dS or ω). Red, positive selection (ω>5); blue, purifying selection (ω = 0); grey, neutral evolution (ω = 1). The proportion of each color represents the fraction of the sequence undergoing the corresponding class of selection. Thick branches indicate statistical support for evolution under episodic diversifying selection as determined by BS-REL. Grey dots denote branches that were tested but not confirmed to be under positive selection using the PAML branch-site models.
(PDF)

Figure S3 Alignment of a TAPBP region and positively selected sites in CTSL2, LNPEP, ERAP1, THOP1, and PSMF1. (A) Multiple alignment of a TAPBP region for a few representative mammalian species. A positively selected site (67S) is colored in red, the cystein residue involved in disulfide-bonding is colored in blue. (B) Ribbon diagram of human CTSL2; sites that define substrate binding are shown in yellow; positively selected sites are in red (whole phylogeny) or green (humans). (C) Schematic representation of LNPEP domains; positively selected sites are indicated in red. (D) Ribbon diagram of ERAP1 with positively selected sites in orange (polymorphic) or green (fixed in humans); the active site is represented in yellow. (E) Ribbon diagram of THOP1 sites subject to positive selection in the human lineage highlighted in green. The active site is shown in yellow. (F) ribbon diagram of PSMF1; the dark grey helix indicates a motif important for protein stability. Positively selected sites are in orange or green depending on their being polymorphic or not, respectively, in humans.
(PDF)

Figure S4 Work-flow and main results for the intra-species analysis. Genes that were defined as targets of positive or balancing selection are shown in red.
(PDF)

Figure S5 Nucleotide diversity estimates for APP genes. π is plotted against θ_W_. The dashed lines represent the 5^th^ and 95^th^ percentiles of a distribution of ∼1000 randomly selected human genes, represented by grey dots.
(PDF)

Figure S6 DIND test and F_ST_ results. (A) The ratio between the ancestral and derived nucleotide diversity, iπ_A_/iπ_D_, is plotted against the derived allele frequency (DAF). The dashed line represents the 95^th^ percentile of a distribution of ∼1000 randomly selected human genes. The grey shaded areas represent frequency ranges where the ratio could not be calculated. (B) F_ST_ values are plotted against the minor allele frequency (MAF). The dashed lines represent the 95^th^ and 99^th^ percentiles of a distribution of SNPs deriving from ∼1000 randomly selected human genes. Black crosses mark SNPs mentioned in the text which display F_ST_ values higher than the 95^th^ percentile.
(PDF)

Figure S7 Analysis of positively selected sites in the *PSME3*/*CNTD1* region. Location of the most likely selection targets in *PSME3*/*CNTD1* region within the UCSC Genome Browser view. Relevant annotation tracks are shown. Variants in green represent both F_ST_ and DIND outliers in AS population.
(PDF)

Figure S8 Extended haplotype homozygosity (EHH) decay plots for variants showing a high lnRsb test.
(PDF)

Figure S9 Overlap between the signals we detected and those identified in previous scans of positive selection. Previously identified regions are represented as black bars and are tagged by author name and population showing selection signatures. The best candidate variants we identified in *NRD1* (upper panel) and *MARCH1* (lower panel) are also shown. Figure S9. Overlap between the signals we detected and those identified in previous scans of positive selection. Previously identified regions are represented as black bars and are tagged by author name and population showing selection signatures. The best candidate variants we identified in *NRD1* (upper panel) and *MARCH1* (lower panel) are also shown.
(PDF)

Figure S10 GENETREE analyses. Estimated haplotype trees for the LD sub-region of *CD207* (A), and for the sequenced regions of *NCF4* (B) and *TAP1* (C). Mutations are represented as black dots and named for their physical position along the region. The absolute frequency of each haplotype is also reported at the bottom of each lineage.
(PDF)

Table S1 List of analysed genes.
(PDF)

Table S2 Average non-synonymous/synonynomus substitution rate ratio (dN/dS).
(PDF)

Table S3 Likelihood ratio test statistics for models of variable selective pressure among sites (F3x4 model of codon frequency).
(PDF)

Table S4 Likelihood ratio test statistics for models of variable selective pressure among sites (F61 model of codon frequency).
(PDF)

Table S5 Likelihood ratio test statistics for branch-site models (*CD207*, *CTSG*, and *CYBB*).
(PDF)

Table S6 SFS-based statistics calculated over whole gene regions using data from the 1000 Genomes Project.
(PDF)

Table S7 Nucleotide diversity and neutrality tests for *CTSB* and *PSMB9* gene regions.
(PDF)

Table S8 Nucleotide diversity and neutrality tests using low coverage 1000 Genomes Project data for the Sanger-resequenced regions.
(PDF)

Table S9 TMRCA estimates.
(PDF)

Table S10 Primer sequences.
(PDF)
