# Supplementary material for: An Evolutionary Analysis of Antigen Processing and Presentation across Different Timescales Reveals Pervasive Selection
Source: PLoS Genet. 2014 Mar 27;10(3):e1004189. doi: 10.1371/journal.pgen.1004189 (PMC3967941; doi:10.1371/journal.pgen.1004189)
Supplement: Figure S1 — Work-flow and main results for the inter-species analysis. Genes that were defined as targets of positive selection are shown in red. (PDF) [file pgen.1004189.s001.pdf]

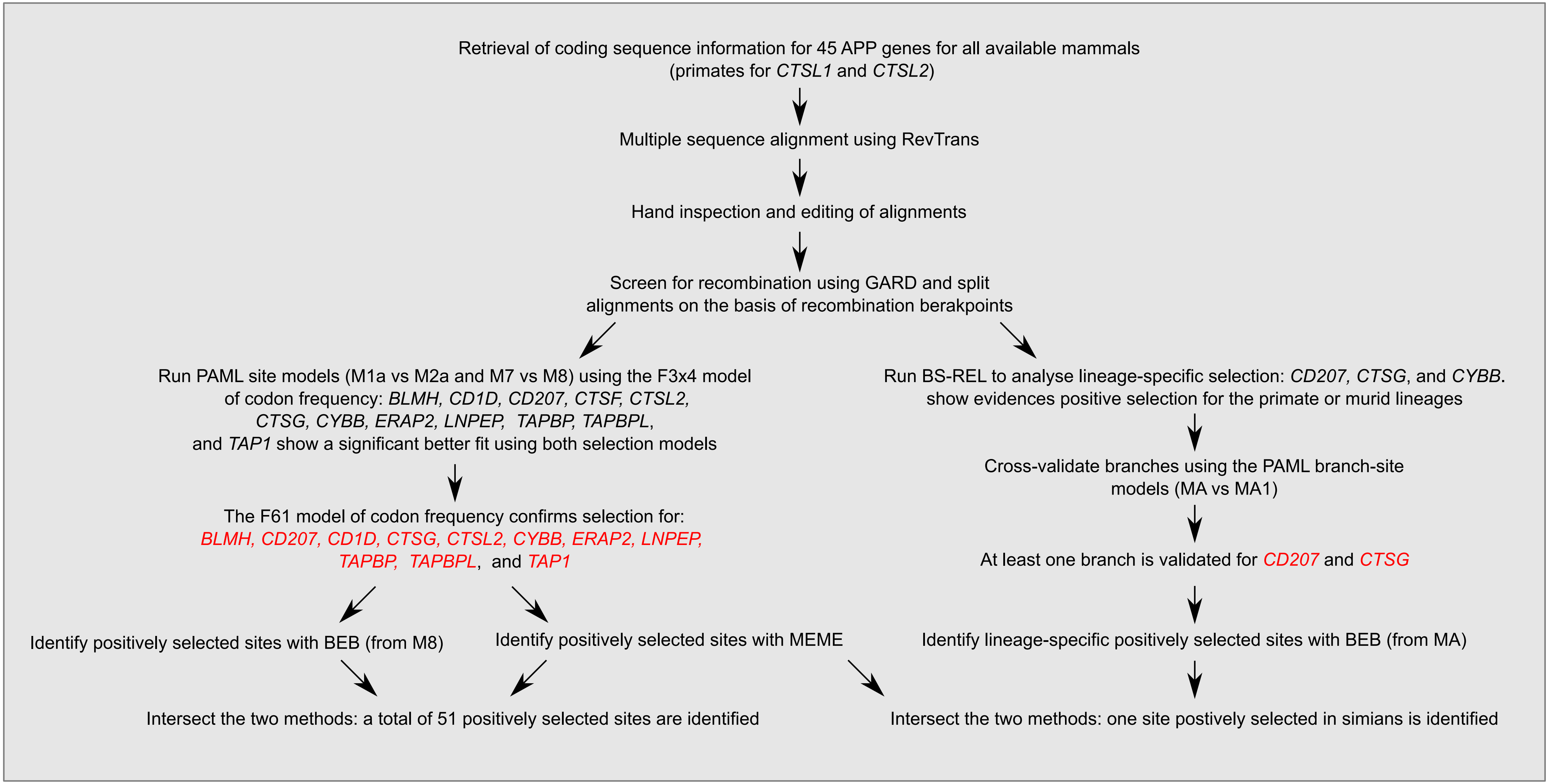
Retrieval of coding sequence information for 45 APP genes for all available mammals
(primates for CTSL1 and CTSL2)
Multiple sequence alignment using RevTrans
Hand inspection and editing of alignments
Screen for recombination using GARD and split alignments on the basis of recombination berakpoints
Run PAML site models (M1a vs M2a and M7 vs M8) using the F3x4 model of codon frequency: BLMH, CD1D, CD207, CTSF, CTSL2, CTSG, CYBB, ERAP2, LNPEP, TAPBP, TAPBPL, and TAP1 show a significant better fit using both selection models
Run BS-REL to analyse lineage-specific selection: CD207, CTSG, and CYBB. show evidences positive selection for the primate or murid lineages
Cross-validate branches using the PAML branch-site models (MA vs MA1)
The F61 model of codon frequency confirms selection for: BLMH, CD207, CD1D, CTSG, CTSL2, CYBB, ERAP2, LNPEP, TAPBP, TAPBPL, and TAP1
At least one branch is validated for CD207 and CTSG
Identify positively selected sites with BEB (from M8)
Identify positively selected sites with MEME
Identify lineage-specific positively selected sites with BEB (from MA)
Intersect the two methods: a total of 51 positively selected sites are identified
Intersect the two methods: one site postively selected in simians is identified
