# Supplementary figures and images for: An Evolutionary Analysis of Antigen Processing and Presentation across Different Timescales Reveals Pervasive Selection
Source: PLoS Genet. 2014 Mar 27;10(3):e1004189. doi: 10.1371/journal.pgen.1004189 (PMC3967941; doi:10.1371/journal.pgen.1004189)

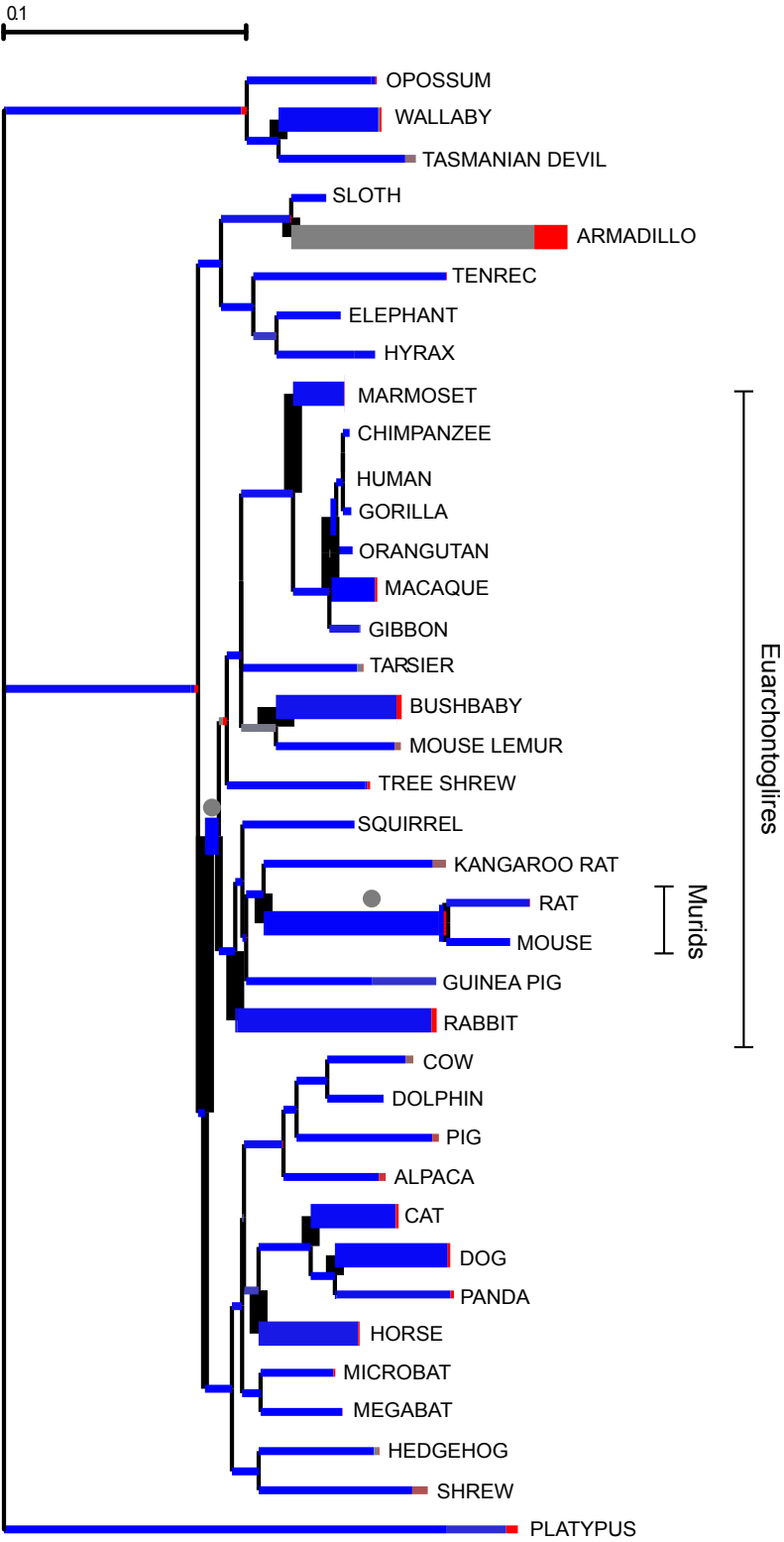

Supplement: Figure S2 — Branch-site analysis of positive selection for CYBB. Branch lengths are scaled to the expected number of substitutions per nucleotide, and branch colors indicate the strength of selection (dN/dS or ω). Red, positive selection (ω>5); blue, purifying selection (ω = 0); grey, neutral evolution (ω = 1). The proportion of each color represents the fraction of the sequence undergoing the corresponding class of selection. Thick branches indicate statistical support for evolution under episodic diversifying selection as determined by BS-REL. Grey dots denote branches that were tested but not confirmed to be under positive selection using the PAML branch-site models. (PDF) [file pgen.1004189.s002.pdf]

**A**

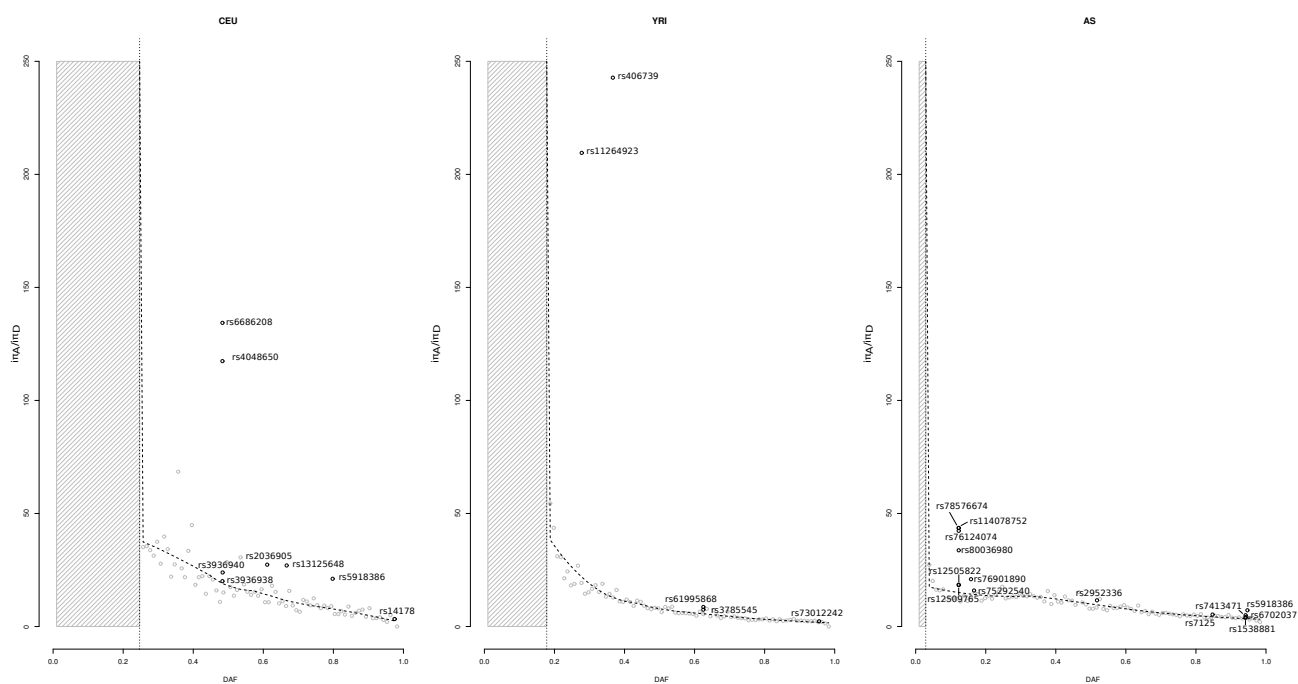

B

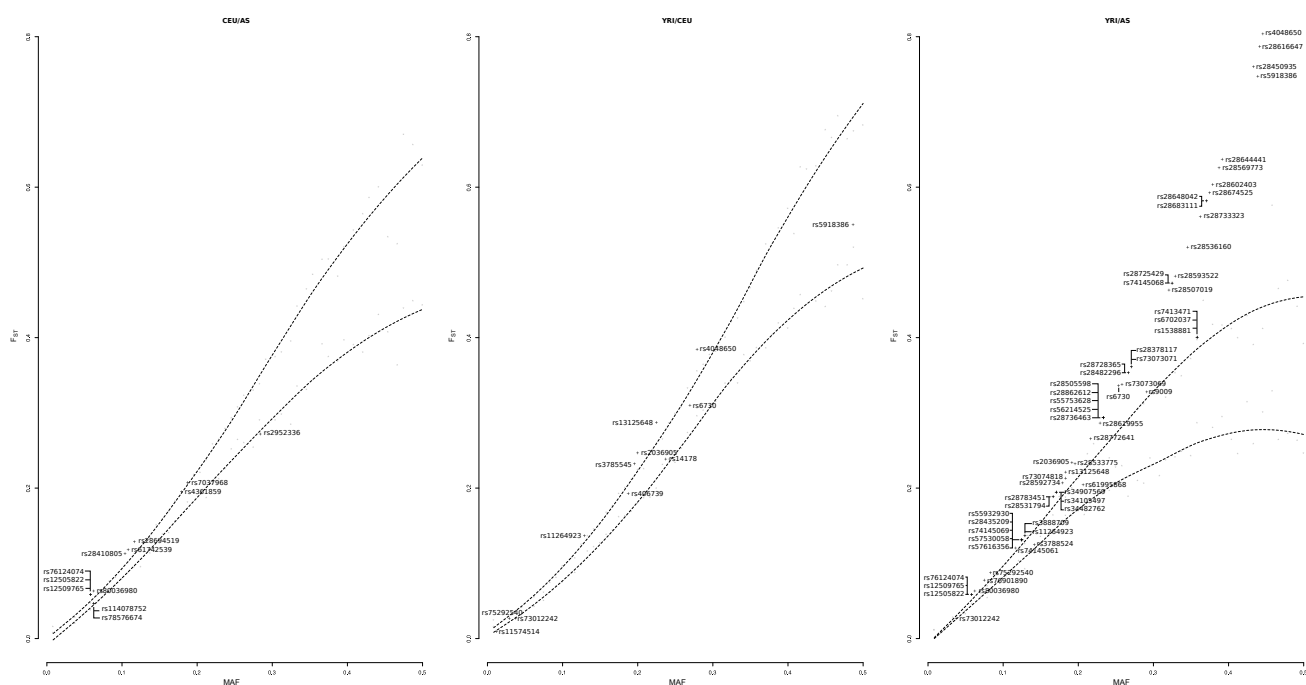

Supplement: Figure S6 — DIND test and FST results. (A) The ratio between the ancestral and derived nucleotide diversity, iπA/iπD, is plotted against the derived allele frequency (DAF). The dashed line represents the 95th percentile of a distribution of ∼1000 randomly selected human genes. The grey shaded areas represent frequency ranges where the ratio could not be calculated. (B) FST values are plotted against the minor allele frequency (MAF). The dashed lines represent the 95th and 99th percentiles of a distribution of SNPs deriving from ∼1000 randomly selected human genes. Black crosses mark SNPs mentioned in the text which display FST values higher than the 95th percentile. (PDF) [file pgen.1004189.s006.pdf]

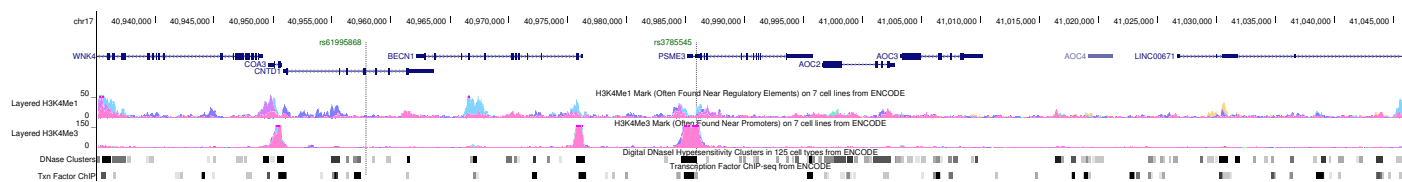

Supplement: Figure S7 — Analysis of positively selected sites in the PSME3/CNTD1 region. Location of the most likely selection targets in PSME3/CNTD1 region within the UCSC Genome Browser view. Relevant annotation tracks are shown. Variants in green represent both FST and DIND outliers in AS population. (PDF) [file pgen.1004189.s007.pdf]

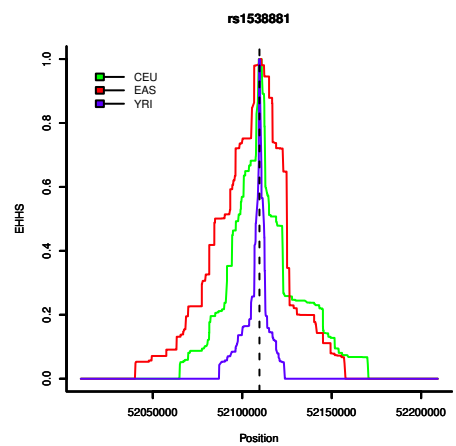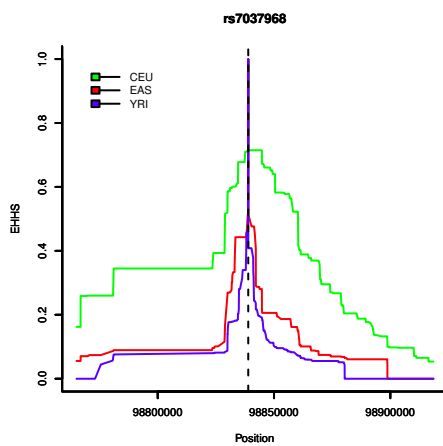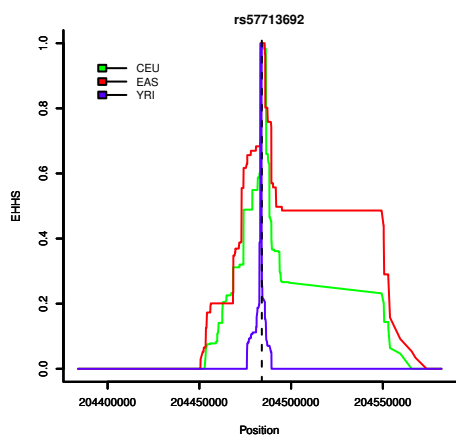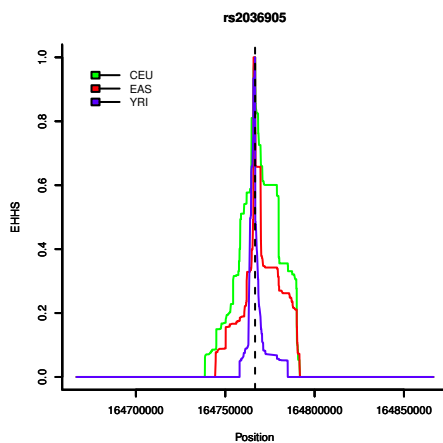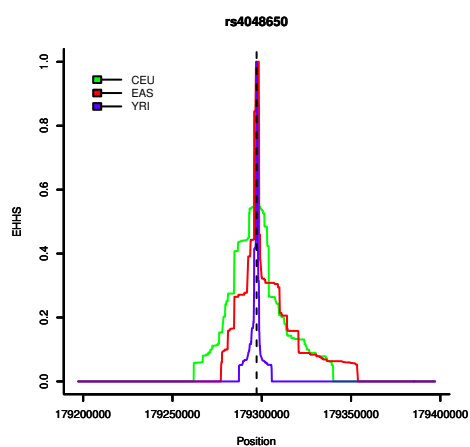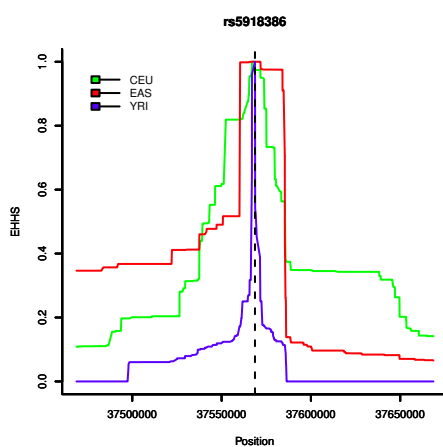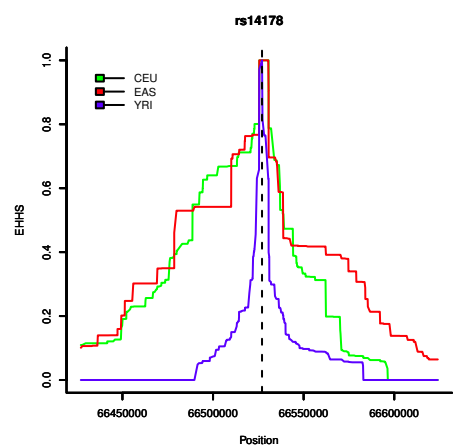

Supplement: Figure S8 — Extended haplotype homozygosity (EHH) decay plots for variants showing a high lnRsb test. (PDF) [file pgen.1004189.s008.pdf]

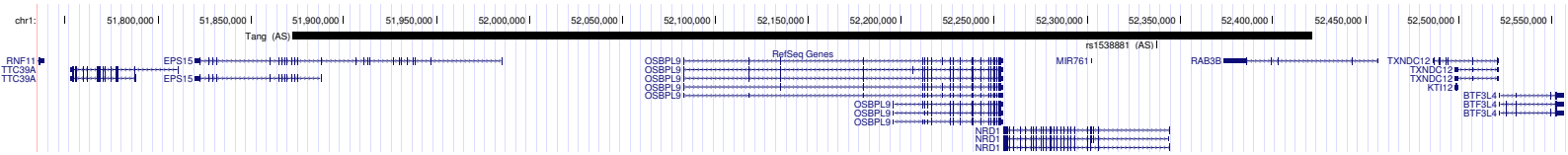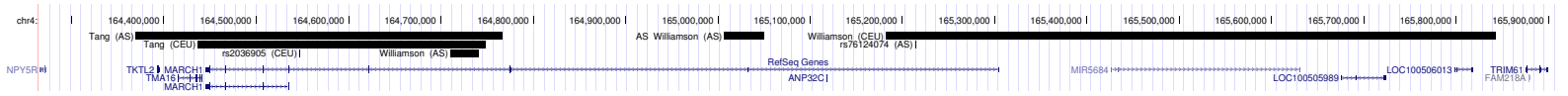

Supplement: Figure S9 — Overlap between the signals we detected and those identified in previous scans of positive selection. Previously identified regions are represented as black bars and are tagged by author name and population showing selection signatures. The best candidate variants we identified in NRD1 (upper panel) and MARCH1 (lower panel) are also shown. Figure S9. Overlap between the signals we detected and those identified in previous scans of positive selection. Previously identified regions are represented as black bars and are tagged by author name and population showing selection signatures. The best candidate variants we identified in NRD1 (upper panel) and MARCH1 (lower panel) are also shown. (PDF) [file pgen.1004189.s009.pdf]

A

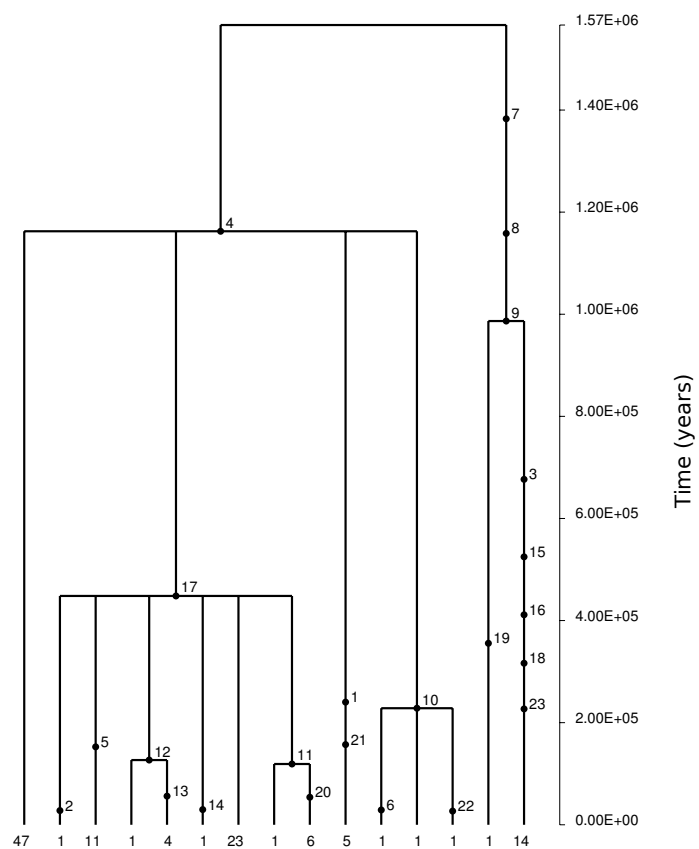

B

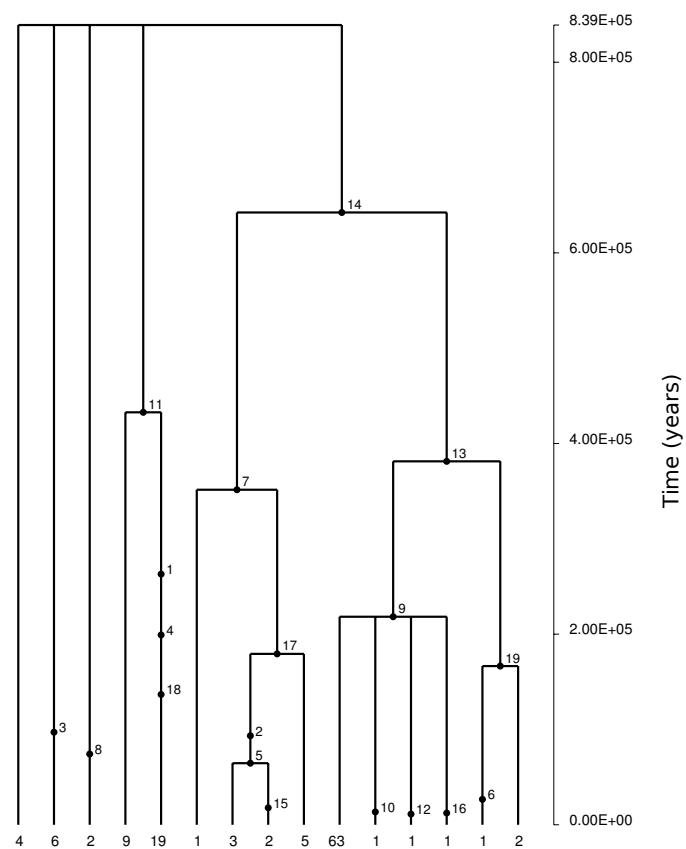

C

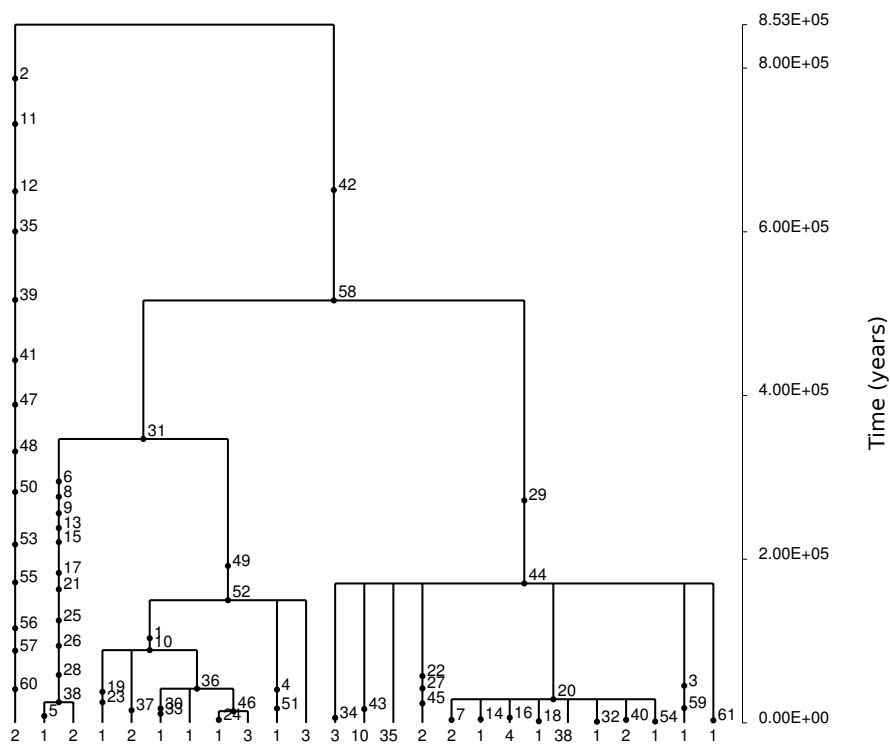

Supplement: Figure S10 — GENETREE analyses. Estimated haplotype trees for the LD sub-region of CD207 (A), and for the sequenced regions of NCF4 (B) and TAP1 (C). Mutations are represented as black dots and named for their physical position along the region. The absolute frequency of each haplotype is also reported at the bottom of each lineage. (PDF) [file pgen.1004189.s010.pdf]
