# Supplementary material for: An Evolutionary Analysis of Antigen Processing and Presentation across Different Timescales Reveals Pervasive Selection
Source: PLoS Genet. 2014 Mar 27;10(3):e1004189. doi: 10.1371/journal.pgen.1004189 (PMC3967941; doi:10.1371/journal.pgen.1004189)
Supplement: Figure S3 — Alignment of a TAPBP region and positively selected sites in CTSL2, LNPEP, ERAP1, THOP1, and PSMF1. (A) Multiple alignment of a TAPBP region for a few representative mammalian species. A positively selected site (67S) is colored in red, the cystein residue involved in disulfide-bonding is colored in blue. (B) Ribbon diagram of human CTSL2; sites that define substrate binding are shown in yellow; positively selected sites are in red (whole phylogeny) or green (humans). (C) Schematic representation of LNPEP domains; positively selected sites are indicated in red. (D) Ribbon diagram of ERAP1 with positively selected sites in orange (polymorphic) or green (fixed in humans); the active site is represented in yellow. (E) Ribbon diagram of THOP1 sites subject to positive selection in the human lineage highlighted in green. The active site is shown in yellow. (F) ribbon diagram of PSMF1; the dark grey helix indicates a motif important for protein stability. Positively selected sites are in orange or green depending on their being polymorphic or not, respectively, in humans. (PDF) [file pgen.1004189.s003.pdf]

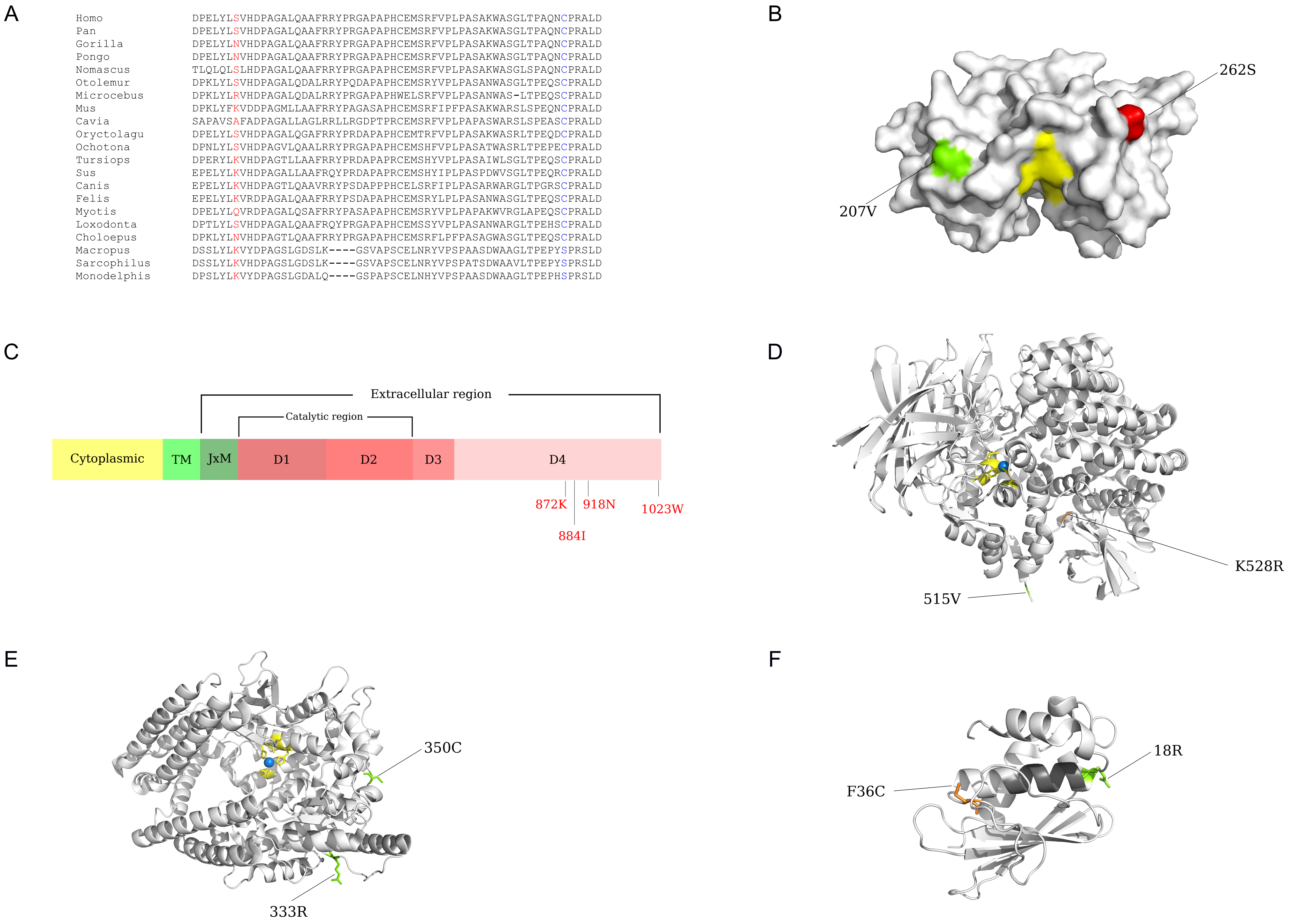

A
Homo DPELYLSVHDPAGALQAAFRRYPRGAPAPHCEMSRFVPLPASAKWASGLTPAQNCPRALD
Pan DPELYLSVHDPAGALQAAFRRYPRGAPAPHCEMSRFVPLPASAKWASGLTPAQNCPRALD
Gorilla DPELYLNVHDPAGALQAAFRRYPRGAPAPHCEMSRFVPLPASAKWASGLTPAQNCPRALD
Pongo DPELYLNVHDPAGALQAAFRRYPRGAPAPHCEMSRFVPLPASAKWASGLTPAQNCPRALD
Nomascus TLQLQLSLHDPAGALQAAFRRYPRGAPAPHCEMSRFVPLPASAKWASGLSPAQNCPRALD
Otolemur DPKLYLSVHDPAGALQDALRRYPQDAPAPHCEMSRYVPLPASANWASGLTPEQSCPRALD
Microcebus DPKLYLRVHDPAGALQDALRRYPRGAPAPHWELSRFVPLPASANWAS-LTPEQSCPRALD
Mus DPKLYFKVDDPAGMLLAAFRRYPAGASAPHCEMSRFIPFPASAKWARSLSPEQNCPRALD
Cavia SAPAVSAFADPAGALLAGLRRLLRGDPTPRCEMSRFVPLPAPASWARSLSPEASCPRALD
Oryctolagu DPELYLSVHDPAGALQGAFRRYPRDAPAPHCEMTRFVPLPASAKWASRLTPEQDCPRALD
Ochotona DPNLYLSVHDPAGVLQAALRRYPRGAPAPHCEMSHFVPLPASATWASRLTPEPECPRALD
Tursiops DPERYLKVHDPAGTLLAAFRRYPRDAPAPRCEMSHYVPLPASAIWLSGLTPEQSCPRALD
Sus EPELYLKVHDPAGALLAAFRQYPRDAPAPRCEMSHYIPLPASPDWVSGLTPEQRCPRALD
Canis EPELYLKVHDPAGTLQAAVRRYPSDAPPPHCELSRFIPLPASARWARGLTPGRSCPRALD
Felis EPELYLKVRDPAGALQAAFRRYPSDAPAPHCEMSRYLPLPASANWASGLTPEQSCPRALD
Myotis DPELYLQVRDPAGALQSAFRRYPASAPAPHCEMSRYVPLPAPAKWVRGLAPEQSCPRALD
Loxodonta DPTLYLSVHDPAGALQAAFRQYPRGAPAPHCEMSSYVPLPASANWARGLTPEHSCPRALD
Choloepus DPKLYLNVHDPAGTLQAAFRRYPRGAPAPHCEMSRFLPFPASAGWASGLTPEQSCPRALD
Macropus DSSLYLKVYDPAGSLGDSLK----GSVAPSCELNRYVPSPAASDWAAGLTPEPYSPRSLD
Sarcophilus DSSLYLKVHDPAGSLGDSLK----GSVAPSCELNRYVPSPATSDWAAVLTPEPYSPRSLD
Monodelphis DPSLYLKVYDPAGSLGDALQ----GSPAPSCELNHYVPSPAASDWAAGLTPEPHSPRSLD
B
262S
207V
C
Extracellular region
Catalytic region
Cytoplasmic
TM
JxM
D1
D2
D3
D4
872K
918N
1023W
884I
D
K528R
515V
E
350C
333R
F
18R
F36C
