# Supplementary material for: An Evolutionary Analysis of Antigen Processing and Presentation across Different Timescales Reveals Pervasive Selection
Source: PLoS Genet. 2014 Mar 27;10(3):e1004189. doi: 10.1371/journal.pgen.1004189 (PMC3967941; doi:10.1371/journal.pgen.1004189)
Supplement: Figure S4 — Work-flow and main results for the intra-species analysis. Genes that were defined as targets of positive or balancing selection are shown in red. (PDF) [file pgen.1004189.s004.pdf]

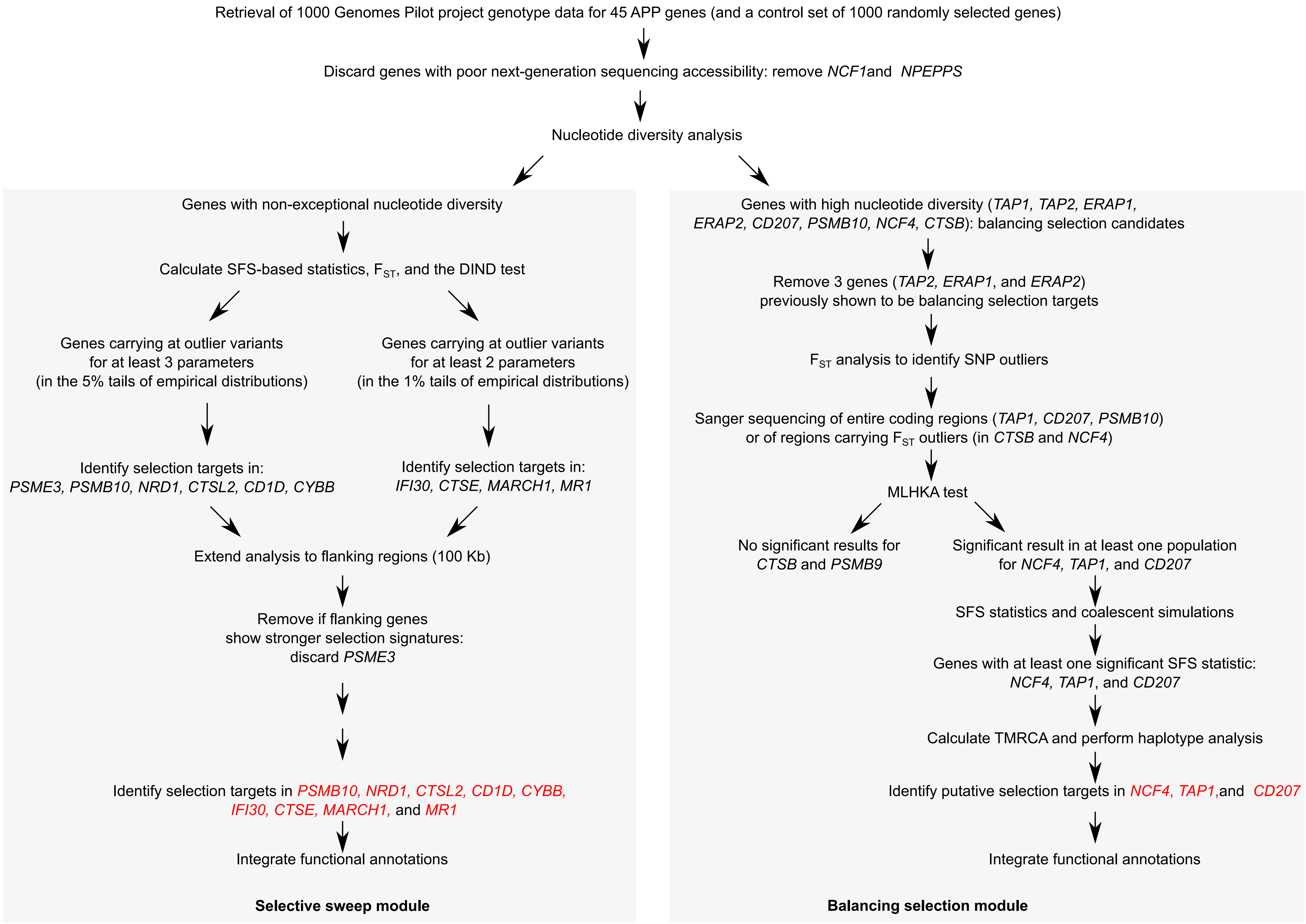
Retrieval of 1000 Genomes Pilot project genotype data for 45 APP genes (and a control set of 1000 randomly selected genes)
Discard genes with poor next-generation sequencing accessibility: remove NCF1and NPEPPS
Nucleotide diversity analysis
Genes with non-exceptional nucleotide diversity
Calculate SFS-based statistics, F_{ST}, and the DIND test
Genes carrying at outlier variants for at least 3 parameters (in the 5% tails of empirical distributions)
Genes carrying at outlier variants for at least 2 parameters (in the 1% tails of empirical distributions)
Identify selection targets in: PSME3, PSMB10, NRD1, CTSL2, CD1D, CYBB
Identify selection targets in: IFI30, CTSE, MARCH1, MR1
Extend analysis to flanking regions (100 Kb)
Remove if flanking genes show stronger selection signatures: discard PSME3
Identify selection targets in PSMB10, NRD1, CTSL2, CD1D, CYBB, IFI30, CTSE, MARCH1, and MR1
Integrate functional annotations
Selective sweep module
Genes with high nucleotide diversity (TAP1, TAP2, ERAP1, ERAP2, CD207, PSMB10, NCF4, CTSB): balancing selection candidates
Remove 3 genes (TAP2, ERAP1, and ERAP2) previously shown to be balancing selection targets
F_{ST} analysis to identify SNP outliers
Sanger sequencing of entire coding regions (TAP1, CD207, PSMB10) or of regions carrying F_{ST} outliers (in CTSB and NCF4)
MLHKA test
No significant results for CTSB and PSMB9
Significant result in at least one population for NCF4, TAP1, and CD207
SFS statistics and coalescent simulations
Genes with at least one significant SFS statistic: NCF4, TAP1, and CD207
Calculate TMRCA and perform haplotype analysis
Identify putative selection targets in NCF4, TAP1,and CD207
Integrate functional annotations
Balancing selection module
