# Supplementary material for: An Evolutionary Analysis of Antigen Processing and Presentation across Different Timescales Reveals Pervasive Selection
Source: PLoS Genet. 2014 Mar 27;10(3):e1004189. doi: 10.1371/journal.pgen.1004189 (PMC3967941; doi:10.1371/journal.pgen.1004189)
Supplement: Table S1 — List of analysed genes. (PDF) [file pgen.1004189.s011.pdf]

Table S1. List of analyzed genes.

Official gene symbol	Chromosomal location (hg19)	Description	Other designations
B2M	chr15:45003684-45010357	beta-2-microglobulin	
BCAP31	chrX:152965946-152990201	B-cell receptor-associated protein 31	BAP31
BLMH	chr17:28575212-28619184	bleomycin hydrolase	
CALR	chr19:13049413-13055304	calreticulin	
CANX	chr5:179125929-179158639	calnexin	major histocompatibility complex class I antigen-binding protein p88
CD1D	chr2:71057342-71062953	CD1d molecule	antigen-presenting glycoprotein CD1d
CD207	chr1:158149736-158156216	CD207 antigen	C-type lectin domain family 4 member K; Langerhans cell specific c-type lectin; langerin
CD74	chr5:149781199-149792332	CD74 antigen	invariant polypeptide of major histocompatibility complex, class II antigen-associated; CLIP
CTSB	chr8:11700033-11725646	cathepsin B	
CTSD	chr11:1773984-1785222	cathepsin D	
CTSE	chr1:206317458-206332104	cathepsin E	
CTSF	chr11:66330934-66336047	cathepsin F	
CTSG	chr14:25042723-25045466	cathepsin G	
CTSL1	chr9:90340973-90346384	cathepsin L1	
CTSL2	chr9:99791958-99801925	cathepsin L2	
CTSS	chr1:150702671-150738433	cathepsin S	
CYBA	chr16:88709696-88717457	cytochrome b-245, alpha polypeptide	
CYBB	chrX:37639269-37672714	cytochrome b-245, beta polypeptide	
ERAP1	chr5:96096513-96149848	endoplasmic reticulum aminopeptidase 1	
ERAP2	chr5:96211643-96255406	endoplasmic reticulum aminopeptidase 2	
IFI30	chr19:18284578-18288927	interferon, gamma-inducible protein 30	gamma-interferon-inducible lysosomal thiol reductase (GILT);
LGMN	chr14:93170151-93215047	legumain	asparaginyl endopeptidase
LNPEP	chr5:96271345-96365115	leucyl/cystinyl aminopeptidase	
MARCH1	chr4:164445449-165304407	membrane-associated ring finger (C3HC4) 1, E3 ubiquitin protein ligase	
MARCH8	chr10:45952816-46090354	membrane-associated ring finger (C3HC4) 8, E3 ubiquitin protein ligase	cellular modulator of immune recognition (c-MIR)
MR1	chr1:181002560-181031074	major histocompatibility complex, class I-related	
NCF1	chr7:74188308-74193602	neutrophil cytosolic factor 1	

NCF2	chr1:183524696-183560056	neutrophil cytosolic factor 2	
NCF4	chr22:37257029-37274059	neutrophil cytosolic factor 4	
NPEPPS	chr17:45608443-45700642	aminopeptidase puromycin sensitive	puromycin-sensitive aminopeptidase, PSA
NRD1	chr1:52254865-52344609	nardilysin (N-arginine dibasic convertase)	
PDIA3	chr15:44038589-44064804	protein disulfide isomerase family A, member 3	ERp57
PSMB10	chr16:67968406-67970780	proteasome (prosome, macropain) subunit, beta type, 10	MECL1
PSMB8	chr6:32808493-32812712	proteasome (prosome, macropain) subunit, beta type, 8 (large multifunctional peptidase 7)	LMP7
PSMB9	chr6:32821937-32827628	proteasome (prosome, macropain) subunit, beta type, 9 (large multifunctional peptidase 2)	LMP2
PSME1	chr14:24605377-24608176	proteasome (prosome, macropain) activator subunit 1 (PA28 alpha)	
PSME2	chr14:24612573-24615855	proteasome (prosome, macropain) activator subunit 2 (PA28 beta)	
PSME3	chr17:40985422-40995777	proteasome (prosome, macropain) activator subunit 3 (PA28 gamma; Ki)	
PSMF1	chr20:1093905-1148426	proteasome (prosome, macropain) inhibitor subunit 1 (PI31)	
TAP1	chr6:32812985-32821748	transporter 1, ATP-binding cassette, sub-family B (MDR/TAP)	
TAP2	chr6:32789609-32806547	transporter 2, ATP-binding cassette, sub-family B (MDR/TAP)	
TAPBP	chr6:33267471-33282164	TAP binding protein (tapasin)	tapasin
TAPBPL	chr12:6561176-6571488	TAP binding protein-like	
THOP1	chr19:2785505-2813599	thimet oligopeptidase 1	
TPP2	chr13:103249285-103331523	tripeptidyl peptidase II	
