# Supplementary material for: An Evolutionary Analysis of Antigen Processing and Presentation across Different Timescales Reveals Pervasive Selection
Source: PLoS Genet. 2014 Mar 27;10(3):e1004189. doi: 10.1371/journal.pgen.1004189 (PMC3967941; doi:10.1371/journal.pgen.1004189)
Supplement: Table S2 — Average non-synonymous/synonynomus substitution rate ratio (dN/dS). (PDF) [file pgen.1004189.s012.pdf]

Table S2. Average non-synonymous/synonynomus substitution rate ratio (dN/dS).

Gene	Number of species	dN/dS (ω)
B2M	33	0.21
BCAP31	32	0.13
BLMH	39	0.12
CALR	34	0.061
CANX	37	0.076
CD1D	28	0.46
CD74	26	0.21
CD207	32	0.47
CTSB	35	0.14
CTSD	20	0.089
CTSE	31	0.20
CTSF	34	0.21
CTSG	28	0.44
CTSL1	11	0.36
CTSL2	11	0.34
CTSS	32	0.23
CYBA	30	0.082
CYBB	38	0.14
ERAP1	34	0.20
ERAP2	26	0.35
IFI30	28	0.25
LGNM	37	0.15
LNPEP	38	0.20
MARCH1	29	0.11
MARCH8	25	0.21
MR1	30	0.40
NCF1	24	0.096
NCF2	32	0.21
NCF4	32	0.14
NPEPPS	32	0.055
NRD1	32	0.13
PDIA3	38	0.11
PSMB8	34	0.16
PSMB9	29	0.12

PSMB10	36	0.13
PSME1	30	0.11
PSME2	31	0.14
PSME3	34	0.070
PSMF1	36	0.21
TAP1	35	0.34
TAP2	26	0.23
TAPBP	33	0.31
TAPBPL	32	0.36
THOP1	30	0.050
TPP2	38	0.049
