# Supplementary material for: An Evolutionary Analysis of Antigen Processing and Presentation across Different Timescales Reveals Pervasive Selection
Source: PLoS Genet. 2014 Mar 27;10(3):e1004189. doi: 10.1371/journal.pgen.1004189 (PMC3967941; doi:10.1371/journal.pgen.1004189)
Supplement: Table S3 — Likelihood ratio test statistics for models of variable selective pressure among sites (F3x4 model of codon frequency). (PDF) [file pgen.1004189.s013.pdf]

Table S3. Likelihood ratio test statistics for models of variable selective pressure among sites (F3x4 model of codon frequency).

Region/selection model (n of codons)	N species	-2ΔLnL	P value (corrected P value)	% of sites (average dN/dS)
B2M (1180)	33			
M1a vs M2a		0	1	
M7 vs M8		43.25	4.05×10^{-10}	6.3 (1.2)
BCAP31 (246)	32			
M1a vs M2a		0	1	
M7 vs M8		5.11	0.077	
BLMH (455)	39			
M1a vs M2a		46.65	7.39×10^{-11} (2.96×10^{-10})	0.8 (3.7)
M7 vs M8		92.81	7.03×10^{-21}(2.81×10^{-20})	0.9 (3.21)
**REG1** (74)				
M1a vs M2a		0	1 (1)	
M7 vs M8		0	1 (1)	
**REG2** (118)				
M1a vs M2a		0	1 (1)	
M7 vs M8		6.52	0.038 (0.15)	1.8 (1.01)
**REG3** (261)				
M1a vs M2a		21.40	2.25×10^{-5} (9.00×10^{-5})	1.1 (2.9)
M7 vs M8		63.33	1.77×10^{-14} (7.08×10^{-14})	1.1 (2.6)
CALR (417)	34			
M1a vs M2a		0	1	
M7 vs M8		7.20	0.027	1.6 (1.0)
CANX (594)	37			
M1a vs M2a		0	1	
M7 vs M8		14.42	7.37×10^{-4}	1.8(1.0)
CD1D (353)	28			
M1a vs M2a		58.60	1.88×10^{-13} (5.64×10^{-13})	3.9 (2.8)
M7 vs M8		66.65	3.65×10^{-15} (1.10×10^{-14})	10.2 (1.9)
**REG1** (153)				
M1a vs M2a		26.97	1.39×10^{-6} (4.17×10^{-6})	5.9 (2.6)
M7 vs M8		35.36	2.10×10^{-8} (6.30×10^{-8})	11.8 (1.9)
**REG2** (200)				
M1a vs M2a		31.61	1.36×10^{-7} (4.08×10^{-7})	3.8 (2.9)
M7 vs M8		37.07	8.91×10^{-9} (2.67×10^{-8})	14.5 (1.7)
CD207 (329)	32			
M1a vs M2a		35.76	1.72×10^{-8}	4.7 (2.3)
M7 vs M8		44.69	1.97×10^{-10}	7.9 (1.7)
CD74 (303)	26			
M1a vs M2a		0.92	0.63	
M7 vs M8		16.41	2.73×10^{-4}	6.4 (1.5)
CTSB (339)	35			
M1a vs M2a		2.38	0.30	
M7 vs M8		21.63	2.01×10^{-5}	2.2 (1.5)

CTSD (390)	20			
M1a vs M2a		5.68	0.058	
M7 vs M8		25.54	2.84×10^{-6}	2.9 (1.5)
CTSE (402)	31			
M1a vs M2a		0	1	
M7 vs M8		14.06	8.88×10^{-4}	11.7 (1.1)
CTSF (483)	34			
M1a vs M2a		9.31	0.0095 (0.0285)	2.0 (2.4)
M7 vs M8		19.93	4.71×10^{-5} (1.41×10^{-4})	4.3 (1.6)
**REG1** (188)				
M1a vs M2a		0	1 (1)	
M7 vs M8		7.86	0.019 (0.057)	5.7 (1.7)
**REG2** (293)				
M1a vs M2a		7.97	0.018 (0.054)	1.8 (2.3)
M7 vs M8		23.23	9.01×10^{-6} (2.73×10^{-5})	7.7 (1.2)
CTSG (255)	28			
M1a vs M2a		83.66	6.80×10^{-19}	9.2 (2.5)
M7 vs M8		85.58	1.58×10^{-19}	14.6 (1.9)
CTSL1 (333)	11			
M1a vs M2a		0.16	0.92	
M7 vs M8		1.04	0.59	
CTSL2 (334)	11			
M1a vs M2a		8.01	0.018	2.9 (3.9)
M7 vs M8		8.47	0.014	3.7 (3.5)
CTSS (335)	32			
M1a vs M2a		0.46	0.79	
M7 vs M8		5.03	0.08	
CYBA (194)	30			
M1a vs M2a		0	1	
M7 vs M8		0	1	
CYBB (570)	38			
M1a vs M2a		205.65	2.02×10^{-45} (8.08×10^{-45})	3.2 (3.3)
M7 vs M8		251.78	2.12×10^{-55} (8.48×10^{-55})	4.5 (2.6)
**REG1** (38)				
M1a vs M2a		23.58	7.57×10^{-6} (3.03×10^{-5})	13.1 (3.4)
M7 vs M8		24.79	4.11×10^{-6} (1.64×10^{-5})	14.1 (2.5)
**REG2** (46)				
M1a vs M2a		0.36	0.83 (1)	
M7 vs M8		9.06	0.011 (0.044)	2.2 (1.3)
**REG3** (480)				
M1a vs M2a		108.73	2.45×10^{-24} (9.80×10^{-24})	2.3 (3.1)
M7 vs M8		161.15	1.01×10^{-35} (4.04×10^{-35})	4.9 (2.1)
ERAP1 (940)	34			
M1a vs M2a		2.68	0.26	
M7 vs M8		19.95	4.66×10^{-5}	6.0 (1.9)

ERAP2 (970)	26			
M1a vs M2a		40.67	$1.47x10^{-9}$ ($5.88x10^{-9}$)	2.2 (2.7)
M7 vs M8		87.37	$1.06x10^{-19}$ ($4.24x10^{-19}$)	5.8 (1.8)
**REG1** (68)				
M1a vs M2a		11.25	0.0036 (0.014)	9.5 (2.8)
M7 vs M8		12.25	0.0022 (0.0088)	11.4 (2.4)
**REG2** (230)				
M1a vs M2a		0	1 (1)	
M7 vs M8		16.49	0.00026 (0.0010)	2.9 (1.9)
**REG3** (670)				
M1a vs M2a		18.04	$1.20x10^{-4}$ ($4.80x10^{-4}$)	2.1 (2,5)
M7 vs M8		53.31	$2.65x10^{-12}$ ($1.06x10^{-11}$)	7.6 (1.7)
IFI30 (247)	28			
M1a vs M2a		0	1	
M7 vs M8		6.40	0.041	6.4 (1.2)
LGNM (433)	37			
M1a vs M2a		0	1	
M7 vs M8		16.05	$3.26x10^{-4}$	3.2 (1.2)
LNPEP (1025)	38			
M1a vs M2a		35.36	$2.10x10^{-8}$ ($6.30x10^{-8}$)	0.3 (3.5)
M7 vs M8		73.96	$8.69x10^{-17}$ ($2.61x10^{-16}$)	5.4 (1.3)
**REG1** (282)				
M1a vs M2a		0	1 (1)	
M7 vs M8		0.58	0.75 (1)	
**REG2** (741)				
M1a vs M2a		43.95	$2.86x10^{-10}$ ($8.58x10^{-10}$)	0.8 (3.1)
M7 vs M8		81.78	$1.75x10^{-18}$ ($5.25x10^{-18}$)	8.9 (1.2)
MARCH1 (289)	29			
M1a vs M2a		0	1	
M7 vs M8		0.26	0.87	
MARCH8 (458)	25			
M1a vs M2a		0	1	
M7 vs M8		7.20	0.027	4.9 (1.3)
MR1 (349)	30			
M1a vs M2a		0	1	
M7 vs M8		21.48	$2.17x10^{-5}$	26.1 (1.0)
NCF1 (399)	24			
M1a vs M2a		0	1	
M7 vs M8		5.34	0.069	
NCF2 (526)	32			
M1a vs M2a		8.88	0.012 (0.6)	0.6 (2.7)
M7 vs M8		21.57	$2.06x10^{-5}$ ($1.03x10^{-4}$)	
**REG1** (212)				
M1a vs M2a		0.73	0.69 (1)	
M7 vs M8		14.37	$7.59x10^{-4}$ ($3.80x10^{-3}$)	1.4 (1.4)
**REG2** (39)				

M1a vs M2a		1.03	0.59 (1)	
M7 vs M8		22.06	1.62×10^{-5} (8.10×10^{-5})	35.7 (1.2)
**REG3** (91)				
M1a vs M2a		0	1 (1)	
M7 vs M8		2.06	0.36 (1)	
**REG4** (181)				
M1a vs M2a		0	1 (1)	
M7 vs M8		2.07	0.35 (1)	
NCF4 (340)	32			
M1a vs M2a		0	1	
M7 vs M8		7.1	0.028	1.6 (1.1)
NPEPPS (834)	32			
M1a vs M2a		0	1	
M7 vs M8		0.98	0.68	
NRD1 (1238)	36			
M1a vs M2a		4.53	0.10	
M7 vs M8		42.38	6.24×10^{-10}	5.6 (1.2)
PDIA3 (505)	38			
M1a vs M2a		0	1	
M7 vs M8		38.71	3.92×10^{-9}	3.4 (1.3)
PSMB8 (276)	34			
M1a vs M2a		0.81	0.66	
M7 vs M8		16.99	2.04×10^{-4}	5.8 (1,2)
PSMB9 (220)	29			
M1a vs M2a		0	1	
M7 vs M8		0.09	0.95	
PSMB10 (220)	29			
M1a vs M2a		0	1	
M7 vs M8		21.01	2.74×10^{-5}	6.7 (1.0)
PSME1 (255)	30			
M1a vs M2a		0	1	
M7 vs M8		7.61	0.022	3.4 (1.3)
PSME2 (254)	31			
M1a vs M2a		95.99	1.43×10^{-21} (5.72×10^{-21})	2.0 (17.5)
M7 vs M8		119.03	1.42×10^{-26} (5.68×10^{-26})	2.7 (16.0)
**REG1** (83)				
M1a vs M2a		0	1 (1)	
M7 vs M8		0.46	0.79 (1)	
**REG2** (27)				
M1a vs M2a		0	1 (1)	
M7 vs M8		0	1 (1)	
**REG3** (126)				
M1a vs M2a		0	1 (1)	
M7 vs M8		3.24	0.19 (0.76)	
PSME3 (252)	34			
M1a vs M2a		0	1	

M7 vs M8		0	1	
PSMF1 (275)	36			
M1a vs M2a		0	1	
M7 vs M8		30.07	$2.95x10^{-7}$	7.1 (1.3)
TAP1 (777)	35			
M1a vs M2a		58.65	$1.83x10^{-13}$ ($5.49x10^{-13}$)	2.8 (2.6)
M7 vs M8		94.36	$3.24x10^{-21}$ ($9.72x10^{-21}$)	8.4 (1.7)
**REG1** (213)				
M1a vs M2a		16.16	$3.10x10^{-4}$ ($9.30x10^{-4}$)	3.3 (2.6)
M7 vs M8		30.76	$2.09x10^{-7}$ ($6.27x10^{-7}$)	14.3 (1.6)
**REG2** (562)				
M1a vs M2a		39.12	$3.12x10^{-9}$ ($9.36x10^{-9}$)	1.6 (2.9)
M7 vs M8		61.72	$3.96x10^{-14}$ ($1.18x10^{-13}$)	6.6 (1.7)
TAP2 (660)	26			
M1a vs M2a		0	1	
M7 vs M8		63.96	$1.29x10^{-14}$	7.9 (1.4)
TAPBP (468)	33			
M1a vs M2a		26.37	$1.87x10^{-6}$	0.9 (3.2)
M7 vs M8		37.72	$6.43x10^{-9}$	1.6 (2.5)
TAPBPL (438)	32			
M1a vs M2a		42.44	$6.06x10^{-10}$	3.0 (2.8)
M7 vs M8		57.84	$2.75x10^{-13}$	6.0 (1.9)
THOP1 (688)	30			
M1a vs M2a		0	1	
M7 vs M8		0.03	0.98	
TPP2 (1262)	38			
M1a vs M2a		0	1	
M7 vs M8		6.55	0.038	11.3 (1.0)

Note: M1a is a nearly neutral model that assumes one ω class between 0 and 1, and one class with ω=1; M2a (positive selection model) is the same as M1a plus an extra class of ω >1. M7 (null model) assumes that 0<ω<1 is beta distributed among sites in 10 classes; M8 (selection model) has an extra class with ω >=1; 2ΔLnL: twice the difference of the natural logs of the maximum likelihood of the models being compared; p value: p value of rejecting the neutral models (M1a or M7) in favor of the positive selection model (M2a or M8); % of sites (average dN/dS): estimated percentage of sites evolving under positive selection by M8 (dN/dS for these codons).
