# Supplementary material for: An Evolutionary Analysis of Antigen Processing and Presentation across Different Timescales Reveals Pervasive Selection
Source: PLoS Genet. 2014 Mar 27;10(3):e1004189. doi: 10.1371/journal.pgen.1004189 (PMC3967941; doi:10.1371/journal.pgen.1004189)
Supplement: Table S4 — Likelihood ratio test statistics for models of variable selective pressure among sites (F61 model of codon frequency). (PDF) [file pgen.1004189.s014.pdf]

Table S4. Likelihood ratio test statistics for models of variable selective pressure among sites (F61 model of codon frequency).

Region/selection model (n of codons)	N species	-2ΔLnL	P value (corrected P value)	% of sites (average dN/dS)
BLMH (455)	39			
M1a vs M2a		54.76	$1.28X10^{-12}$ ($5.12X10^{-12}$)	0.8 (3.3)
M7 vs M8		93.88	$4.11X10^{-21}$ ($1.64X10^{-20}$)	0.6 (4.3)
**REG1** (74)				
M1a vs M2a		0	1 (1)	
M7 vs M8		0	1 (1)	
**REG2** (118)				
M1a vs M2a		0	1 (1)	
M7 vs M8		0	1 (1)	
**REG3** (261)				
M1a vs M2a		23.67	$7.25X10^{-6}$ ($2.90X10^{-5}$)	1.0 (3.0)
M7 vs M8		62.72	$2.39X10^{-14}$ ($9.56X10^{-14}$)	1.1 (2.7)
CD207 (329)	32			
M1a vs M2a		32.17	$1.04X10^{-7}$	5.1 (1.2)
M7 vs M8		41.97	$7.71X10^{-10}$	8.8 (1.7)
CD1D (353)	28			
M1a vs M2a		54.09	$1.79X10^{-12}$ ($5.37X10^{-12}$)	6.7 (2.3)
M7 vs M8		68.07	$1.65X10^{-15}$ ($4.95X10^{-15}$)	11.2 (1.9)
**REG1** (153)				
M1a vs M2a		19.99	$4.55X10^{-5}$ ($1.36X10^{-4}$)	8.2 (2.1)
M7 vs M8		29.11	$4.77X10^{-7}$ ($1.43X10^{-6}$)	15.3 (1.7)
**REG2** (200)				
M1a vs M2a		32.35	$9.42X10^{-8}$ ($2.82X10^{-7}$)	6.2 (2.4)
M7 vs M8		40.53	$1.58X10^{-9}$ ($4.74X10^{-9}$)	9.0 (2.1)
CTSG (255)	28			
M1a vs M2a		92.10	$9.99X10^{-21}$	13.3 (2.3)
M7 vs M8		106.74	$6.63X10^{-24}$	17.2 (1.9)
CTSL2 (334)	11			
M1a vs M2a		6.19	0.045	3.3 (3.2)
M7 vs M8		6.58	0.037	5.0 (2.8)
CYBB (570)	38			
M1a vs M2a		158.03	$4.82X10^{-35}$ ($1.93X10^{-34}$)	3.0 (2.9)
M7 vs M8		201.18	$2.06X10^{-44}$ ($8,24^{-44}$)	5.0 (2.1)
**REG1** (38)				
M1a vs M2a		5.80	0.055 (0.22)	
M7 vs M8		4.63	0.098 (0.39)	
**REG2** (46)				
M1a vs M2a		0.07	0.97 (1)	
M7 vs M8		8.99	0.011 (0.044)	2.1 (1.1)

**REG3** (480)				
M1a vs M2a		102.42	$5.75X10^{-23}$ ($2.30X10^{-22}$)	3.2 (2.6)
M7 vs M8		157.57	$6.07X10^{-35}$ ($2.43X10^{-34}$)	5.1 (2.0)
ERAP2 (970)	26			
M1a vs M2a		36.04	$1.49X10^{-8}$ ($5.96X10^{-8}$)	1.9 (2.7)
M7 vs M8		76.99	$1.91X10^{-17}$ ($4.76X10^{-17}$)	
**REG1** (68)				
M1a vs M2a		6.07	0.048 (0.19)	8.8 (2.4)
M7 vs M8		7.14	0.028 (0.11)	11.5 (2.1)
**REG2** (230)				
M1a vs M2a		0	1 (1)	
M7 vs M8		11.88	0.003 (0.012)	1.7 (2.0)
**REG3** (670)				
M1a vs M2a		16.15	$3.1X10^{-4}$ ($1.24X10^{-3}$)	1.6 (2.6)
M7 vs M8		45.97	$1.04X10^{-10}$ ($4.16X10^{-10}$)	
LNPEP (1025)	38			
M1a vs M2a		33.18	$6.23X10^{-8}$ ($1.89X10^{-7}$)	0.4 (3.2)
M7 vs M8		69.68	$7.38X10^{-16}$ ($2.21X10^{-15}$)	5.7 (1.3)
**REG1** (282)				
M1a vs M2a		0	1 (1)	
M7 vs M8		1.14	0.56 (1)	
**REG2** (741)				
M1a vs M2a		40.96	$1.27X10^{-9}$ ($1.89X10^{-9}$)	0.7 (3.1)
M7 vs M8		73.50	$1.09X10^{-16}$ ($3.27X10^{-16}$)	9.4 (1.2)
TAP1 (777)	35			
M1a vs M2a		56.02	$6.85X10^{-13}$ ($2.06X10^{-12}$)	3.4 (2.4)
M7 vs M8		90.39	$2.35X10^{-20}$ ($7.05X10^{-20}$)	8.3 (1.7)
**REG1** (213)				
M1a vs M2a		12.51	0.0019 (0.0057)	3.9 (2.2)
M7 vs M8		22.59	$1.24X10^{-5}$ ($3.72X10^{-5}$)	8.7 (1.7)
**REG2** (562)				
M1a vs M2a		27.84	$9.01X10^{-7}$ ($2.70X10^{-6}$)	2.5 (2.2)
M7 vs M8		56.96	$4.28X10^{-13}$ ($1.28X10^{-12}$)	8.2 (1.5)
TAPBP (468)	33			
M1a vs M2a		41.56	$9.41X10^{-10}$	1.9 (3.1)
M7 vs M8		55.10	$1.08X10^{-12}$	2.6 (2.4)
TAPBPL (438)	32			
M1a vs M2a		37.74	$6.37X10^{-9}$	1.5 (3.4)
M7 vs M8		39.36	$2.84X10^{-9}$	2.1 (2.5)

Note: M1a is a nearly neutral model that assumes one ω class between 0 and 1, and one class with ω=1; M2a (positive selection model) is the same as M1a plus an extra class of ω >1. M7 (null model) assumes that 0<ω<1 is beta distributed among sites in 10 classes; M8 (selection model) has an extra class with ω >=1; 2ΔLnL: twice the difference of the natural logs of the maximum likelihood of the models being compared; p

value: p value of rejecting the neutral models (M1a or M7) in favor of the positive selection model (M2a or M8); % of sites (average dN/dS): estimated percentage of sites evolving under positive selection by M8 (dN/dS for these codons).
