# Supplementary material for: An Evolutionary Analysis of Antigen Processing and Presentation across Different Timescales Reveals Pervasive Selection
Source: PLoS Genet. 2014 Mar 27;10(3):e1004189. doi: 10.1371/journal.pgen.1004189 (PMC3967941; doi:10.1371/journal.pgen.1004189)
Supplement: Table S5 — Likelihood ratio test statistics for branch-site models (CD207, CTSG, and CYBB). (PDF) [file pgen.1004189.s015.pdf]

Table S5. Likelihood ratio test statistics for models of positive selection on specific branches (F3x4 codon frequency model)

Gene	Foreground branch[a]	Degree of freedom	-2ΔLnL[b]	p value[c]	FDR-corrected p value[d]	Positively selected sites[e]
CD207	Murids	1	7.81	0.0052	0.010	-
	Rodents	1	0.002	0.964	0.964	-
CTSG	Great apes	1	6.65	0.010	0.015	-
	Simians	1	16.07	$6.1x10^{-5}$	$1.8x10^{-4}$	175I
	Euarchontoglires	1	0	1	1	-
CYBB	Murids	1	0	1	1	-
	Euarchontoglires	1	0	1	1	-

[a] MA and MA1 are branch-site models that assume four classes of sites: the MA model allows a proportion of codons to have $\omega \geq 1$ on the foreground branches (those to be tested for selection), whereas the MA1 model does not.
[b] 2ΔLnL: twice the difference of the natural logs of the maximum likelihood of the models being compared.
[c] p value of rejecting the neutral model in favor of the positive selection model.
[d] False discovery rate (FDR)-corrected p value
[e] Positively selected sites identified through BEB (from MA model, cut-off= 0.90)
