# Supplementary material for: An Evolutionary Analysis of Antigen Processing and Presentation across Different Timescales Reveals Pervasive Selection
Source: PLoS Genet. 2014 Mar 27;10(3):e1004189. doi: 10.1371/journal.pgen.1004189 (PMC3967941; doi:10.1371/journal.pgen.1004189)
Supplement: Table S6 — SFS-based statistics calculated over whole gene regions using data from the 1000 Genomes Project. (PDF) [file pgen.1004189.s016.pdf]

Table S6. SFS-based statistics calculated over whole gene regions using data from the 1000 Genomes Project.

Gene	Chrom	P[a]	S[b]	H[c]		Tajima's D		Fu & Li's D*		Fu & Li's F*	
				value	rank[d]	value	rank[d]	value	rank[d]	value	rank[d]
B2M	chr15	YRI	16	0.36	0.64	-0.38	0.19	0.01	**0.04**	-0.16	0.06
		CEU	13	-0.83	0.29	-1.07	**0.05**	1.50	0.78	0.68	0.33
		AS	9	-0.46	0.44	0.36	0.21	1.31	0.32	1.16	0.24
BCAP31	chrX	YRI	51	-0.93	0.9	-1.22	**0.01**	0.66	0.14	-0.12	**0.05**
		CEU	7	-1.53	0.15	-0.1	0.20	1.2	0.60	0.89	0.41
		AS	7	-0.91	0.31	-0.96	**0.04**	0.33	0.07	-0.12	**0.04**
BLMH	chr17	YRI	118	0.80	0.90	-0.58	0.13	0.37	0.09	-0.05	0.07
		CEU	77	0.04	0.59	-0.28	0.17	0.08	0.14	-0.09	0.12
		AS	46	-0.32	0.48	0.48	0.24	1.93	0.68	1.60	0.41
CALR	chr19	YRI	15	0.10	0.50	0.49	0.67	1.58	0.61	1.41	0.71
		CEU	4	1.53	>0.99	0.08	0.26	0.93	0.45	0.77	0.37
		AS	2	1.68	>0.99	1.16	0.44	0.67	0.11	0.96	0.18
CANX	chr5	YRI	70	-0.32	0.28	0.09	0.43	1.29	0.39	0.94	0.39
		CEU	58	-0.79	0.30	1.32	0.73	0.40	0.22	0.94	0.44
		AS	49	-1.21	0.26	2.18	0.81	1.25	0.30	1.97	0.58
CD1D	chr1	YRI	15	-3.79	**0.01**	-0.52	0.14	-0.10	**0.03**	-0.30	**0.04**
		CEU	11	-2.45	0 .06	2.03	0.92	1.41	0.74	1.95	0.89
		AS	14	0.27	0.69	2.83	0.93	0.96	0.18	1.96	0.58
CD207	chr2	YRI	47	-0.08	0.38	0.04	0.39	1.20	0.34	0.87	0.34
		CEU	27	-0.73	0.31	-0.09	0.21	1.91	**0.95**	1.35	0.64
		AS	21	-0.18	0.52	0.71	0.31	1.77	0.57	1.63	0.42
CD74	chr5	YRI	36	-0.72	0.14	-0.67	0.10	0.84	0.19	0.28	0.13
		CEU	23	-0.83	0.29	-0.36	0.15	1.40	0.73	0.86	0.40
		AS	13	-1.26	0.25	0.86	0.34	1.50	0.41	1.52	0.37
CTSB	chr8	YRI	176	0.25	0.58	0.33	0.57	1.69	0.67	1.30	0.63
		CEU	122	0.31	0.72	1.57	0.81	0.77	0.36	1.35	0.64
		AS	68	-0.27	0.49	2.62	0.91	1.43	0.38	2.33	0.77
CTSD	chr11	YRI	42	-0.86	0.11	-0.60	0.12	1.87	0.81	1.03	0.45
		CEU	34	-1.60	0.15	-0.40	0.15	1.08	0.53	0.58	0.29
		AS	38	-0.07	0.57	0.05	0.14	1.80	0.59	1.31	0.29
CTSE	chr1	YRI	71	0.13	0.51	-0.22	0.26	0.42	0.10	0.18	0.12
		CEU	31	-1.10	0.23	0.27	0.32	-1.08	**0.04**	-0.65	0.06
		AS	8	0.44	0.76	-1.39	**0.02**	-1.24	**0.01**	-1.53	**0.01**
CTSF	chr11	YRI	20	0.54	0.75	-0.54	0.14	1.28	0.39	0.71	0.27
		CEU	13	0.21	0.67	1.31	0.72	1.50	0.78	1.71	0.81
		AS	16	0.54	0.80	1.56	0.58	1.62	0.48	1.91	0.55
CTSG	chr14	YRI	14	0.12	0.51	-0.04	0.34	0.96	0.23	0.71	0.27

		CEU	9	-0.62	0.35	0.97	0.59	-0.24	0.08	0.22	0.18
		AS	7	-0.03	0.58	1.63	0.61	1.18	0.25	1.58	0.40
CTSL1	chr9	YRI	16	-0.46	0.22	0.45	0.64	1.08	0.29	1.01	0.44
		CEU	12	-0.29	0.46	0.14	0.28	1.46	0.77	1.18	0.56
		AS	8	-0.71	0.37	0.67	0.29	-0.41	**0.03**	-0.04	**0.05**
CTSL2	chr9	YRI	33	0.76	0.88	1.29	0.93	1.69	0.67	1.84	0.93
		CEU	18	-3.90	**0.02**	0.46	0.40	0.69	0.33	0.72	0.34
		AS	17	0.65	0.83	3.04	**0.96**	1.14	0.24	2.21	0.72
CTSS	chr1	YRI	50	0.47	0.71	1.63	**0.97**	1.75	0.72	2.04	**0.97**
		CEU	69	-0.64	0.34	0.96	0.58	0.36	0.20	0.74	0.36
		AS	45	0.61	0.82	2.75	0.93	1.66	0.50	2.53	0.86
CYBA	chr16	YRI	41	0.93	0.94	1.07	0.90	1.02	0.25	1.26	0.60
		CEU	27	-0.70	0.32	3.17	**>0.99**	1.91	**0.95**	2.88	**>0.99**
		AS	27	-0.80	0.35	1.06	0.41	1.91	0.67	1.89	0.53
CYBB	chrX	YRI	72	0.96	0.95	-0.34	0.09	1.00	0.24	0.54	0.19
		CEU	33	-2.11	0.8	-0.31	016	0.46	0.23	0.19	0.17
		AS	14	-4.92	**0.01**	-1.81	**0.01**	-0.13	**0.04**	-0.87	**0.02**
ERAP1	chr5	YRI	244	0.48	0.71	0.84	0.83	2.18	**0.97**	1.89	0.94
		CEU	262	-0.20	0.49	1.15	0.66	2.17	**0.99**	2.05	0.92
		AS	150	0.23	0.68	2.40	0.87	2.42	**0.97**	2.89	**0.96**
ERAP2	chr5	YRI	211	0.04	0.46	2.24	**>0.99**	2.14	**0.95**	2.63	**100**
		CEU	217	-0.13	0.52	2.53	**0.98**	1.44	0.76	2.31	**96**
		AS	162	-0.37	0.47	3.72	**>0.99**	2.28	0.90	3.50	**>0.99**
IFI30	chr19	YRI	16	-1.270	0.06	1.14	0.91	0.54	0.12	0.92	0.38
		CEU	10	-1.33	0.19	2.43	**0.97**	0.64	0.31	1.51	0.72
		AS	12	-5.06	**0.01**	-0.15	0.12	1.46	0.39	1.05	0.20
LGMN	chr14	YRI	180	-0.48	0.21	-0.55	0.13	1.48	0.53	0.69	0.27
		CEU	93	-0.27	0.46	1.15	0.66	-0.07	0.11	0.55	0.28
		AS	88	-0.06	0.57	1.03	0.40	2.01	0.73	1.90	0.54
LNPEP	chr5	YRI	258	0.35	0.63	0.81	0.82	1.49	0.53	1.42	0.72
		CEU	233	-0.91	0.26	1.28	0.71	1.47	0.78	1.66	0.79
		AS	195	-0.27	0.49	1.76	0.66	2.30	0.91	2.47	0.84
MARCH1	chr4	YRI	3664	3.44	>0.99	0.44	0.64	1.62	0.63	1.29	0.63
		CEU	2745	3.31	>0.99	0.45	0.40	1.26	0.63	1.06	0.49
		AS	2389	4.42	>0.99	1.62	0.60	2.43	**0.98**	2.46	0.83
MARCH8	chr10	YRI	415	-2.67	**0.01**	-0.61	0.12	2.07	0.93	1.02	0.45
		CEU	410	-3.12	**0.03**	-0.58	0.12	1.81	0.92	0.86	0.40
		AS	419	-2.68	0.09	-0.41	0.09	2.50	**0.99**	1.41	0.33
MR1	chr1	YRI	99	0.59	0.78	0.45	0.64	1.69	0.67	1.38	0.69
		CEU	75	-0.19	0.49	0.62	0.46	1.54	0.81	1.38	0.65
		AS	60	-0.44	0.44	1.67	0.62	1.89	0.66	2.16	0.69

NCF2	chr1	YRI	112	-0.40	0.24	1.14	0.91	1.45	0.50	1.58	0.82
		CEU	89	-1.44	0.17	0.84	0.54	1.28	0.64	1.31	0.62
		AS	62	-1.90	0.16	2.02	0.76	2.31	0.92	2.63	0.90
NCF4	chr22	YRI	107	0.50	0.73	0.72	0.78	2.01	0.90	1.74	0.89
		CEU	69	-0.31	0.45	1.25	0.69	1.27	0.63	1.52	0.73
		AS	62	-0.55	0.42	1.17	0.44	1.92	0.67	1.92	0.55
NRD1	chr1	YRI	268	-0.75	0.14	-0.59	0.12	1.59	0.61	0.73	0.28
		CEU	190	-1.33	0.19	-0.27	0.17	0.50	0.25	0.19	0.18
		AS	106	-3.76	**0.03**	-1.63	**0.01**	1.00	0.18	-0.18	**0.05**
PDIA3	chr15	YRI	39	0.94	0.95	0.99	0.89	1.53	0.56	1.58	0.82
		CEU	32	-1.41	0.18	0.07	0.26	1.00	0.48	0.76	0.36
		AS	25	-0.34	0.47	1.67	0.62	0.68	0.12	1.27	0.27
PSMB8	chr6	YRI	17	-0.52	0.19	-1.14	**0.02**	1.65	0.65	0.73	0.28
		CEU	16	-0.43	0.41	-0.76	0.09	0.01	0.13	-0.34	0.09
		AS	14	-0.16	0.53	0.70	0.30	0.37	0.08	0.59	0.11
PSMB9	chr6	YRI	33	-0.48	0.21	0.71	0.78	2.02	0.91	1.79	0.91
		CEU	29	0.03	0.58	0.51	0.41	0.16	0.16	0.36	0.22
		AS	24	0.16	0.65	1.98	0.74	1.44	0.39	1.97	0.58
PSMB10	chr16	YRI	5	0.06	0.47	1.38	0.94	1.02	0.25	1.35	0.67
		CEU	2	-2.70	**0.05**	-0.36	0.15	0.67	0.32	0.41	0.23
		AS	1	1.33	0.99	0.28	0.19	0.48	0.08	0.49	0.09
PSME1	chr14	YRI	5	0.47	0.71	-0.93	0.06	-0.06	0.33	-0.41	**0.02**
		CEU	2	0.06	0.60	-1.35	**0.01**	-2.90	**>0.01**	-2.83	**0.01**
		AS	0	NA		NA		NA		NA	
PSME2	chr14	YRI	8	0.62	0.80	-0.85	0.06	-0.41	**0.02**	-0.66	**0.2**
		CEU	3	-0.80	0.30	-0.09	0.21	0.81	0.37	0.62	0.30
		AS	3	0.65	0.83	0.96	0.38	0.81	0.13	1.01	0.19
PSME3	chr17	YRI	19	0.53	0.75	-1.02	**0.03**	-0.20	**0.03**	-0.61	**0.02**
		CEU	3	0.14	0.63	-1.43	**0.02**	-2.06	**0.01**	-2.19	**0.01**
		AS	6	-3.19	0.06	-1.31	**0.02**	0.13	0.06	-0.42	**0.03**
PSMF1	chr20	YRI	211	0.22	0.56	-0.27	0.24	1.67	0.66	0.96	0.41
		CEU	144	0.48	0.79	0.54	0.42	1.06	0.52	1.00	0.46
		AS	97	-0.62	0.40	1.25	0.47	1.94	0.69	1.97	0.58
TAP1	chr6	YRI	74	-0.67	0.16	-0.56	0.13	1.70	0.68	0.89	0.36
		CEU	76	-1.07	0.23	-1.45	**0.02**	1.22	0.61	0.09	0.15
		AS	33	-1.17	0.26	0.40	0.21	1.37	0.35	1.17	0.24
TAP2	chr6	YRI	159	-1.28	0.06	1.22	0.93	2.18	**0.97**	2.11	**0.98**
		CEU	137	-1.39	0.18	1.14	0.65	1.98	**0.96**	1.93	0.88
		AS	113	-0.27	0.49	1.97	0.74	2.29	0.91	2.58	0.88
TAPBP	chr6	YRI	44	1.18	0.99	0.43	0.64	1.12	0.31	1.01	0.44
		CEU	30	0.85	0.93	0.98	0.59	-0.47	0.07	0.13	0.16

		AS	27	0.10	0.63	0.99	0.39	0.78	0.13	1.04	0.20
TAPBPL	chr12	YRI	32	-0.16	0.35	0.24	0.52	1.00	0.24	0.84	0.32
		CEU	26	0.76	0.91	2.44	**0.97**	1.50	0.78	2.24	**0.95**
		AS	23	1.21	0.98	2.90	0.94	1.40	0.36	2.37	0.79
THOP1	chr19	YRI	134	0.50	0.73	0.16	0.46	1.35	0.43	0.99	0.42
		CEU	54	-0.01	0.56	2.23	**0.95**	0.48	0.24	1.46	0.70
		AS	56	0.77	0.87	3.40	**0.99**	1.84	0.62	2.99	**0.97**
TPP2	chr13	YRI	342	3.91	>0.99	1.45	**0.95**	1.77	0.73	1.96	**0.95**
		CEU	309	4.60	>0.99	2.06	0.92	1.71	0.89	2.24	**0.95**
		AS	252	5.48	>0.99	3.07	**0.96**	2.43	**0.98**	3.26	**>0.99**

[a] population
[b] number of segregating sites
[c] normalized Fay and Wu's H
[d] percentile rank relative to a distribution of ~1000 randomly selected human genes
