# Supplementary material for: An Evolutionary Analysis of Antigen Processing and Presentation across Different Timescales Reveals Pervasive Selection
Source: PLoS Genet. 2014 Mar 27;10(3):e1004189. doi: 10.1371/journal.pgen.1004189 (PMC3967941; doi:10.1371/journal.pgen.1004189)
Supplement: Table S7 — Nucleotide diversity and neutrality tests for CTSB and PSMB9 gene regions. (PDF) [file pgen.1004189.s017.pdf]

Table S7. Nucleotide diversity and neutrality tests for *CTSB* and *PSMB9* gene regions.

**Gene**	**L**[a]	**Pop**[b]	**S**[c]	θ_w [d]		π[f]		**MLHKA**	
				**value**	**rank**[e]	**value**	**rank**[e]	**K**[g]	**p**[h]
CTSB	4.6	YRI	58	29.58	**>0.99**	26.77	**0.99**	1.38	0.66
		CEU	30	15.30	**0.98**	23.98	**0.99**	0.97	0.17
		AS	22	11.22	0.94	13.02	0.94	0.79	0.21
PSMB9	5.6	YRI	31	13.00	0.86	16.26	**0.95**	1.96	0.25
		CEU	27	11.32	0.92	14.41	0.93	2.00	0.32
		AS	26	10.91	0.94	15.04	0.94	2.17	0.077

[a] length of analyzed resequenced region (in kb);
[b] population;
[c] number of segregating sites;
[d] θ_W estimation per site (x 10^{-4});
[e] percentile rank relative to a distribution of 238 5 kb windows from NIEHS genes;
[f] π estimation per site (x 10^{-4});
[g] selection parameter (k>1 indicates an excess of polymorphism compared to divergence; k< 1 indicates the opposite situation)
[h] *p* values obtained by applying a calibrated population genetics model, as described in the text
