# Supplementary material for: An Evolutionary Analysis of Antigen Processing and Presentation across Different Timescales Reveals Pervasive Selection
Source: PLoS Genet. 2014 Mar 27;10(3):e1004189. doi: 10.1371/journal.pgen.1004189 (PMC3967941; doi:10.1371/journal.pgen.1004189)
Supplement: Table S8 — Nucleotide diversity and neutrality tests using low coverage 1000 Genomes Project data for the Sanger-resequenced regions. (PDF) [file pgen.1004189.s018.pdf]

Supplementary Table S8. Nucleotide diversity and neutrality tests using low coverage 1000 Genomes Project data for the Sanger-resequenced regions

Gene	L[a]	Pop[b]	θw [c]			π [g]			Tajima's D			Fu and Li's D*			Fu and Li's F*			Recombination rate
			value[d]	rank[e]	rank[f]	value[d]	rank[e]	rank[f]	value[d]	rank[e]	rank[f]	value[d]	rank[e]	rank[f]	value[d]	rank[e]	rank[f]	cM/Mb
CD207	4.7	YRI	16.63	0.99	0.98	17.91	0.97	0.98	0.23	0.57	0.7	1.06	0.52	0.85	0.87	0.54	0.82	2.205
		CEU	10.26	0.96	0.96	10.21	0.87	0.89	-0.02	0.35	0.42	1.89	0.99	0.78	1.37	0.76	0.64	2.013
		AS	7.89	0.95	0.90	9.85	0.89	0.81	0.7	0.41	0.45	1.74	0.96	0.96	1.61	0.71	0.82	0.883
NCF4	3.0	YRI	9.9	0.87	0.94	12.89	0.9	0.94	0.81	0.79	0.68	1.62	0.88	0.34	1.58	0.88	0.43	2.750
		CEU	8.66	0.93	0.95	15.17	0.96	0.88	2.01	0.89	0.40	0.96	0.62	0.21	1.6	0.84	0.24	0.975
		AS	7.42	0.93	0.96	12.89	0.94	0.97	1.9	0.79	0.89	1.46	0.79	>0.99	1.93	0.85	0.99	0.105
TAP1	7.2	YRI	15.47	0.98	0.96	12.98	0.9	0.76	-0.51	0.23	0.12	1.65	0.88	0.97	0.88	0.54	0.79	0
		CEU	16.52	0.998	0.72	8.98	0.82	0.56	-1.45	0.02	0.3	1.2	0.75	0.34	0.078	0.26	0.31	0
		AS	7.27	0.93	0.77	8.23	0.83	0.74	0.4	0.33	0.5	1.37	0.75	0.41	1.17	0.47	0.42	1.45

[a] length of analyzed resequenced region (in kb);
[b] population;
[c] θ_w estimation per site (x 10^{-4});
[d] values have been calculated using 1000 Genomes Pilot Project data
[e] 1000 Genomes Project data: percentile rank relative to a distribution of 5 kb windows from ~1000 randomly selected human genes ;
[f] Sanger sequencing data: percentile rank relative to a distribution of 238 5 kb windows from NIEHS genes;
[g] π estimation per site (x 10^{-4});
