# Supplementary material for: An Evolutionary Analysis of Antigen Processing and Presentation across Different Timescales Reveals Pervasive Selection
Source: PLoS Genet. 2014 Mar 27;10(3):e1004189. doi: 10.1371/journal.pgen.1004189 (PMC3967941; doi:10.1371/journal.pgen.1004189)
Supplement: Table S9 — TMRCA estimates. (PDF) [file pgen.1004189.s019.pdf]

Table S9. TMRCA estimates.

	Evans et al. TMRCA (SD[a])	Network TMRCA (SD[a])	ρ[b]	GENETREE TMRCA (SD[a])	Ne[c]	n[d]
CD207	1.02 My (462 Ky)	1.92 My[e] (601 Ky)	6.59	1.57 My[e] (384 Ky)	18800	6
NCF4	1.56 My (711 Ky)	1.79 My (553 Ky)	5.53	0.84 My[e] (260 Ky)	12973	4
TAP1	0.66 My (337 Ky)	1.67 My (438 Ky)	9.75	0.85 My (147 Ky)	2022	10

[a] standard deviation.
[b] average pairwise difference between haplotypes.
[c] effective population size.
[d] GENETREE assumes an infinite-site model without recombination, sites that violate these assumptions were removed: n indicates the number of sites removed.
[e] TMRCA was calculated over a shorter region of LD
