# Supplementary material for: An Evolutionary Analysis of Antigen Processing and Presentation across Different Timescales Reveals Pervasive Selection
Source: PLoS Genet. 2014 Mar 27;10(3):e1004189. doi: 10.1371/journal.pgen.1004189 (PMC3967941; doi:10.1371/journal.pgen.1004189)
Supplement: Table S10 — Primer sequences. (PDF) [file pgen.1004189.s020.pdf]

Table S10. List of primers used to perform PCR amplifications and resequencing analysis

Gene	Primer	Nucleotide sequence
CD207	CD207-F1	GAAAGCTCAGACTTCTGAGG
	CD207-seqF	CATGTGCAGTAACAGGCTGTG
	CD207-seqR1	GATCAATGTTTCCTGAGGAC
	CD207-seqR	GGATCTGGGATTGAGAAAGTC
	CD207-F2	ACTTAGGGGCTGTTAGGAGA
	CD207-R1	CAAAGAGTGTCTTCCCCATG
	CD207-seqF2a	GAGGATGGGCAAGATTATAC
	CD207-seqF2b	ACCATTCTCTTGCGGTCCGTG
	CD207-seqR2a	CATTCCAGCTGCCTCCAAGA
	CD207-R2	GAGACTGCCTTTCCCACATA
CTSB	CTSB-F1	GTTTCACCGTGTTGCCAAGGC
	CTSB-seqR	GCAATCATTCCACTGTCCTGC
	CTSB-seqF1a	TTGATCTAGCATCTGGTTC
	CTSB-seqF1b	CAGGCATATTGGTGAGTGCCT
	CTSB-F2	CAGGACTGGCACGACAGGC
	CTSB-R1	GACAGGATCACTGTGGAATCG
	CTSB-seqF2	GCTGCTCCAGGTACACCCTG
	CTSB-seqR2	CCACCCGAGAGCCTGGTTG
	CTSB-R2	AGGGAGTGGCGTGTCCTGG
NCF4	NCF4-F3	GCAGGTAAGGTTAGGCTGAGAT
	NCF4-SEQR3	GCCTCTGTAAGAGCCCTGAGT
	NCF4-F4	GATCTCGGCTCACTGCAACCT
	NCF4-R3	CAGTAGTTCGAGACCAGCCTG
	NCF4-R4	GTAGTGAGTCCCTGAGAGTGG
PSMB9	PSMB9-F1	TCTCGGAAAGTCCCAGGAACA
	PSMB9-seqF1a	TGGCCCAGATGCTGCCTTACT
	PSMB9-seqR1a	AGTAGTCCAGAGCAGAAGCCA
	PSMB9-seqF1b	AGGAATGGAGTTGACCTTCC
	PSMB9-seqR1b	GATGGAAAGAAGACCCTCAA
	PSMB9-F2	GTGCCTGTAATCCCAGCTAT
	PSMB9-R1	AGGGAGTCAACAGTCACCAAG
	PSMB9-seqF2a	TTCCAGGAGCTGGACACTG
	PSMB9-seqR2a	TGGGTCACAATATCACCTTC
	PSMB9-seqF2b	AGAACTGGAGGAACCTCCACT
	PSMB9-R2	TCAGTACGTGATCACCTGTC

TAP1	TAP1-F1	GAGGTAACACACTCAAGGCA
	TAP1-seqF1	GTAAAGAGATGAGCATTCAAG
	TAP1-F2	TCTGACGGTTGTAGCTGGAT
	TAP1-R1	TGGCTGCAGTGGGACAAGAG
	TAP1-seqF2	GTAGATGGAGAGCAGTACC
	TAP1-seqR2	GAATCACACTGGGGAGTGA
	TAP1-R2	TCTCCTGACCTTGTGATCCG
	TAP1-F3	CTGGCTTCCACTATTCCCAT
	TAP1-seqF3	TGAGCCTCAGGTTGCTAGGA
	TAP1-seqR3	ATACAGGTTCTCTAGGGAAC
	TAP1-F4	CAGGAGAAACCTGTCTGGTT
	TAP1-R3	CTTTATAGTGCAGTGCTGGAG
	TAP1-seqF4	TGTTAGAGATGAGGATGCCC
	TAP1-R4	CTGATTTCCACGCTTGCTAC
