# Supplementary material for: An Evolutionary Analysis of Antigen Processing and Presentation across Different Timescales Reveals Pervasive Selection
Source: PLoS Genet. 2014 Mar 27;10(3):e1004189. doi: 10.1371/journal.pgen.1004189 (PMC3967941; doi:10.1371/journal.pgen.1004189)
Supplement: Figure S5 — Nucleotide diversity estimates for APP genes. π is plotted against θW. The dashed lines represent the 5th and 95th percentiles of a distribution of ∼1000 randomly selected human genes, represented by grey dots. (PDF) [file pgen.1004189.s005.pdf]

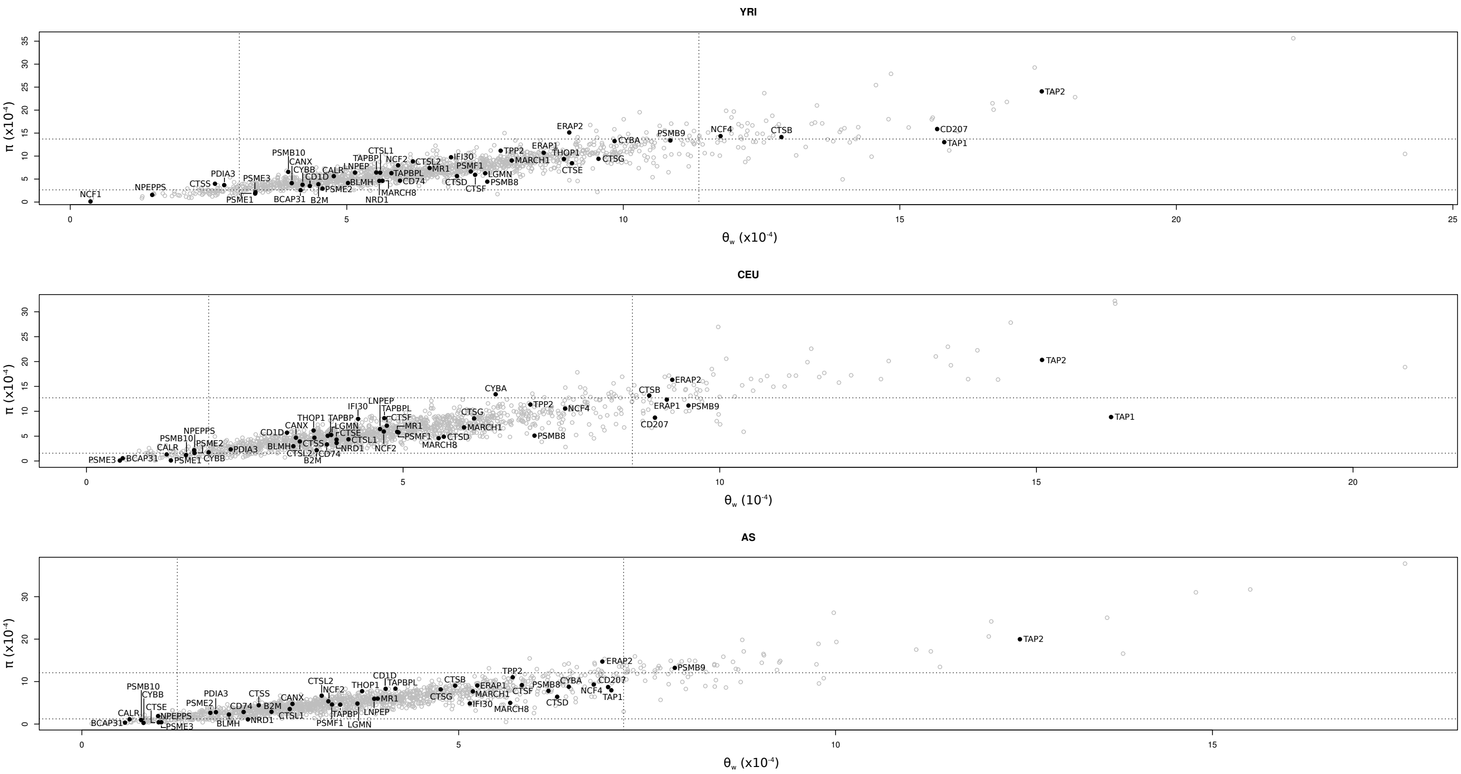
YRI
π (x10⁻⁴)
θw (x10⁻⁴)
0
5
10
15
20
25
30
35
CEU
π (x10⁻⁴)
θw (10⁻⁴)
AS
π (x10⁻⁴)
θw (x10⁻⁴)
